# A pH‐Switchable Triple Hydrogen‐Bonding Motif

**DOI:** 10.1002/open.201900338

**Published:** 2020-01-08

**Authors:** Heather M. Coubrough, Barbora Balonova, Christopher M. Pask, Barry A. Blight, Andrew J. Wilson

**Affiliations:** ^1^ School of Chemistry and Astbury Centre for Structural Molecular Biology University of Leeds Woodhouse Lane Leeds LS2 9JT U.K; ^2^ Department of Chemistry University of New Brunswick Toole Hall, Fredericton NB E3B 5A3 Canada

**Keywords:** hydrogen bonding, pH-responsive systems, self-sorting networks, supramolecular chemistry, molecular recognition

## Abstract

A stimuli responsive linear hydrogen bonding motif, capable of *in situ* protonation and deprotonation, has been investigated. The interactions of the responsive hydrogen bonding motif with complementary partners were examined through a series of ^1^H NMR experiments, revealing that the recognition preference of the responsive hydrogen bonding motif in a mixture can be switched between two states.

## Graphical abstract

**A pH responsive** linear triple hydrogen bonding motif capable of protonation and deprotonation, has been investigated. A series of ^1^H NMR experiments, revealed that the preference of this responsive hydrogen bonding motif in a mixture of hydrogen bonding motifs, can be switched between two self‐sorted states.

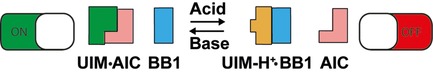

Significant effort in supramolecular chemistry is directed towards use of narcissistic (self‐loving) and/or social (self‐loathing) self‐sorting^1^, ^2^, ^3^ molecular recognition motifs^4^, ^5^ and assemblies^6^, ^7^ for development of systems that change architecture and component usage^8^, ^9^, ^10^, ^11^, ^12^, ^13^ in response to chemical^14^, ^15^, ^16^ or physical stimuli.^17^, ^18^ Although ammonium crown‐ether hydrogen‐bonding interactions for self‐sorting have been explored,^19^, ^20^, ^21^ the majority of such systems exploit shape and geometrical complementarity of metal‐ligand interactions. Self‐sorting systems using weaker interactions, such as hydrogen‐bonding set within the context of linear arrays of donors (*D*) and acceptors (*A*) in hydrogen‐bonding motifs (HBMs),^22^, ^23^, ^24^, ^25^, ^26^, ^27^, ^28^, ^29^ have received less attention. The absence of HBMs from the toolkit of supramolecular synthons used to generate systems which can transition between different self‐sorted configurations arises because: (i) the requirements for high‐fidelity recognition may seem at odds with the need for promiscuous recognition required for transition between different self‐sorted states, (ii) HBMs that change recognition behavior in response to stimuli are sparse.^30^, ^31^, ^32^, ^33^, ^34^, ^35^, ^36^, ^37^ Previously, our group demonstrated that a selection of HBMs could be used to create systems where successive addition of components changed the self‐sorted configuration (where at least one component exhibits promiscuous, and other components, selective recognition behavior),^38^ leading upon introduction of further components, to a self‐sorting network.^39^ The inability to switch recognition preference in a stimuli dependent manner remains a limitation of such a system. To develop truly switchable self‐sorting networks thus requires access to stimuli responsive HBMs. Herein, we describe the design and ^1^H NMR study of a responsive HBM capable of switching recognition preference towards complementary HBMs, in response to protons.

HBM ureidoimidazole (UIM) was identified as a potential responsive motif; the crystal structure of a synthetic intermediate (***I***) revealed protonation of the imidazole resulting in a donor‐ donor (*DD*) array (Figure 1a,b) (See ESI Scheme 2 for synthesis of UIM **1**). We reasoned that protonation and deprotonation of UIM **1** would result in switching between the donor‐donor‐acceptor (*DDA*) array of neutral UIM **1** and the donor‐donor‐donor (*DDD*) array of protonated UIM‐H^+^ **1‐H^+^** (Figure 1c). In turn it was anticipated that this responsive hydrogen bonding motif would interact preferentially with different complementary hydrogen bonding motifs depending on the conditions. Neutral UIM **1** (*DDA* array) had in prior work been shown to interact with AIC **2** (*AAD* array)^40^, ^41^, ^42^ whereas protonated UIM‐H^+^ **1‐H^+^** (*DDD* array) was expected to interact with a complementary *AAA* array such as BB1 **3** (Figure 1d,e). Charge reinforced hydrogen bonding motifs have previously been reported,^43^, ^44^, ^45^ whilst Leigh and co‐workers demonstrated switching ‘on’ and ‘off’ for cation supported hydrogen‐bonding interactions of linear arrays using hydrogen iodide (HI) and 1,8‐Diazabicyclo[5.4.0]undec‐7‐ene (DBU).^36^, ^46^

**Figure 1:**
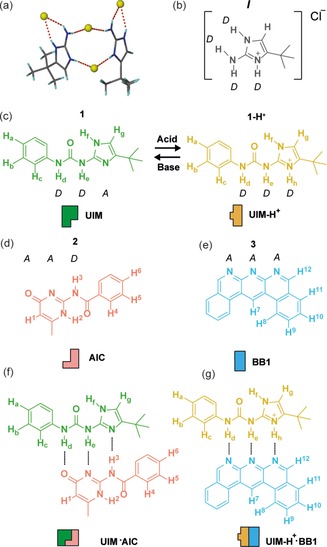
(a) Crystal structure of intermediate ***I***, (b) chemical structure of intermediate ***I***, (c) chemical structure and schematic showing protonation and deprotonation between **1** and **1‐H^+^**, (d) chemical structure and schematic of AIC **2**, (e) chemical structure and schematic of BB1 **3**, (f) chemical and schematic structure of UIM ⋅ AIC **1** ⋅ **2** and (g) chemical and schematic structure of UIM**‐**H^+^.BB1 **1‐H^+^** ⋅ **3**.

^1^H NMR in deuterochloroform was used to study switching between neutral and protonated HBMs, **1** and **1‐H^+^** respectively (Figure 2 and Figure ESI 1). Switching was initially assessed using hydrochloric acid for protonation and sodium hydrogen carbonate for deprotonation (condition A). On addition of excess 4 M hydrochloric acid in 1,4‐dioxane to UIM **1** (10 mM) distinct changes in chemical shifts were observed to the spectrum of UIM **1** (Figure ESI 1). Resonances H_a_, H_b_, H_c_ and H_g_ (between 6 and 8 ppm) were observed to shift downfield on the addition of hydrochloric acid (Figure ESI 1c,d). In addition, the NH resonances H_d_, H_e_, H_f_ and H_h_ (between 9 and 13 ppm), which are broadened for UIM **1** (under neutral conditions), can be seen in the ^1^H NMR spectra of **1‐H^+^** Figure ESI 1c). The changes in chemical shift and sharpening of these specific resonances, relative to UIM **1**, indicate a change in chemical environment and protonation of the nitrogen atom in the aminoimidazole ring (at position H_f_), in a similar manner to that observed in the crystal structure of intermediate ***I***, to generate UIM‐HCl **1‐H^+^**. ^1^H NMR titration was carried out to determine the equivalents of acid required to protonate UIM **1**. At 5 mM of UIM **1** in chloroform 0.25, 0.5, 0.75, 1 and 3 equivalents of 4 M HCl in 1,4‐dioxane were added (see Figure ESI 2). This demonstrated that significant changes in chemical shift of the aromatic resonances (H_a_, H_b_, H_c_) occurred even at 0.5 equivalents of hydrochloric acid. With 1 equivalent of hydrochloric acid the broad NH resonances of UIM sharpened, indicating complete protonation of the imidazole. Protonation was reversed by addition of sodium hydrogen carbonate to regenerate neutral UIM **1** see Figure ESI 1b. After filtration of the salts, the ^1^H NMR spectrum of the resulting solution revealed broadening of the NH resonances (H_d_, H_e_, H_f_ and H_h_) and an upfield change in chemical shift of the aromatic resonances (H_a_, H_b_, H_c_ and H_g_) matching that observed for the neutral species (Figure ESI 1b,d). The switching process can be cycled by the addition of acid and base respectively (Figure ESI 1a). After four cycles, the resolution of the NH resonances in the ^1^H NMR spectrum of **1‐H^+^** was diminished, likely due to the concentration of the sample decreasing slightly with each cycle in part through loss of sample during filtration. To further prolong the recyclability of switching, and identify conditions where intermediate filtration can be avoided, a further set of reagents‐trifluoroacetic acid (TFA) and 1,4‐diazabicyclo[2.2.2]octane (DABCO) were considered (condition B). Under these conditions, however, it was necessary to add excess base to ensure complete deprotonation, which made spectral interpretation more challenging (Figure ESI 3). Moreover, the presence of TFA appeared to subtly influence the spectra at higher concentrations, possibly due to interaction of the anion with the HBM. We explored several additional weaker acids and bases e. g. camphor sulfonic acid and 1,8‐Diazabicyclo[5.4.0]undec‐7‐ene (not shown); whilst similar spectral changes were observed, the quality and practicality of handling, led us to use conditions A and B in subsequent studies.

**Figure 2:**
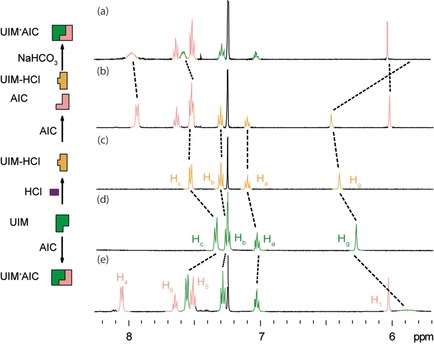
Self‐sorting behavior of proton responsive UIM **1** (condition A) with AIC **2** studied by ^1^H NMR (500 MHz, 5 mM, CDCl_3_) (a) UIM⋅AIC **1** ⋅ **2** formed after washing UIM‐HCl **1‐H^+^** and AIC **2** mixture with NaHCO_3_, (b) UIM‐HCl **1‐H^+^** (protonated with 4 M HCl in 1,4‐dioxane) and AIC **2**, (c) UIM‐HCl **1‐H^+^** (protonated with 4 M HCl in 1,4‐dioxane), (d) UIM **1**, (e) UIM⋅AIC **1** ⋅ **2** and (f) AIC **2**. Refer to Figure 1 for the resonance assignment and chemical structure.

The capacity of UIM **1** and UIM **1‐H^+^** to interact with complementary HBMs was tested by the addition of AIC **2**, bearing an *AAD* array. The heterodimerisation of UIM **1** and AIC **2** through triple hydrogen bonding has been well studied,^41^ and can be identified by a diagnostic downfield shift of H_a_, H_b_ and H_c_ resonances together with an upfield shift and broadening of resonance H_g_ of UIM **1** in the ^1^H NMR and downfield shift of resonance H_4_ of AIC **2** (Figure 2d–f). This interaction can be turned ‘on’ by deprotonation of UIM **1‐H^+^** using condition A. Starting with UIM **1** (Figure 2d), addition of hydrochloric acid gave UIM‐HCl **1‐H^+^** (Figure 2c). Upon addition of AIC **2** to UIM‐HCl **1‐H^+^** no significant spectral changes were observed for UIM‐HCl **1‐H^+^** and AIC **2** and the spectra were consistent with those observed for the individual components e. g. sharp H_g_ and H_1_ resonances of **1** and **2** respectively (Figure 2b). The complementary *ADD‐DAA* interaction was thus prevented as a consequence of protonation giving a *DDD* and *DAA* pair that do not interact. The interaction can be turned on by addition of sodium hydrogen carbonate to give the UIM⋅AIC **1** ⋅ **2** complex (Figure 2a). Here, the H_g_ resonance of **1** was broadened and shifted upfield as expected and the spectrum aligned well with that observed for UIM⋅AIC **1** ⋅ **2** (Figure 2e). Although, the ^1^H NMR spectrum in Figure 2a,e align thus indicating the presence of the UIM⋅AIC **1** ⋅ **2** heterodimer, there are subtle differences; likely arising due to a change in compound concentration during the filtration cycle as noted before. Overall this series of ^1^H NMR experiments revealed proton dependent switching whereby AIC **2** can complex with neutral UIM **1** but not protonated UIM‐HCl **1‐H^+^**. Switching ‘off’ and ‘on’ behavior for the **1** ⋅ **2** intermolecular interaction was also observed using condition B (see Figure ESI 4).

To further explore the recognition behavior of the switchable HBM **1**, a complementary HBM **3** (BB1 **3** with *AAA* character) was added to create a three‐component system. On the addition of BB1 **3** to **1** ⋅ **2**, the heterodimer remained intact and BB1 **3** showed no interaction‐imposed perturbations in the ^1^H NMR spectra (Figure 3d–e). As expected UIM **1** favored the matched triple hydrogen bonding interaction with AIC **2** over the mismatched array of BB1 **3**. This can be understood through the comparison of the binding constants for UIM ⋅ AIC **1** ⋅ **2** dimer versus UIM ⋅ BB1 **1** ⋅ **3** dimer. The K_a_ of the **1** ⋅ **2** dimer, previously reported at 3.3×10^4^ M^−1^ in deuterochloroform (Table 1), is a degree of magnitude larger than that determined for the **1** ⋅ **3** dimer by NMR titration in this study (*K*_a_=2×10^3^ M^−1^, see Table 1 and Figure ESI 6 & 7 for titration data).^47^, ^48^ Hence it is expected that UIM **1** interacts with AIC **2** preferentially over BB1 **3**. However, when BB1 **3** was added to a mixture of AIC **2** and UIM‐HCl **1‐H^+^** an interaction was observed between BB1 **3** and UIM‐HCl **1‐H^+^**. ^1^H NMR spectra (Figure 3c,b) showed changes in the chemical shifts of H_a_, H_b_, H_c_ and H_g_ resonances correlating to UIM‐HCl⋅BB1 **1‐H^+^** ⋅ **3** interaction, whereas the chemical shifts of AIC **2** were unaffected in comparison to an isolated sample. This highlighted the preference for UIM‐HCl **1‐H^+^** to hydrogen bond with BB1 **3** rather than AIC **2**. Consistent with these observations, the association constant for the UIM‐HCl⋅BB1 **1‐H^+^** ⋅ **3** interaction was determined to be *K*_a_≥1×10^5^ M^−1^ by NMR titration in deuterochloroform, which is stronger than the **1** ⋅ **2** dimer (Table 1 and Figure ESI 8 & 9 for titration data).^47^, ^48^ The UIM‐HCl⋅BB1 **1‐H^+^** ⋅ **3** interaction in the presence of AIC **2** could be switched to favor the UIM⋅AIC **1** ⋅ **2** complex in the presence of BB1 **3** by washing with basic sodium hydrogen carbonate solution (Figure 3a). A similar series of ^1^H NMR experiments carried out using condition B mirrored this behavior; here the switching was less effective, presumably arising as a consequence of the lower basicity of DABCO in comparison to sodium hydrogen‐carbonate in combination with an increased p*K*_a_ of the **1‐H^+^** when hydrogen‐bonded in the **1‐H^+^** ⋅ **3** complex (Figure ESI 5). Overall these experiments demonstrated the ability of UIM **1** to undergo proton responsive switching in a mixture and hence switch recognition preference between UIM⋅AIC **1** ⋅ **2** and UIM‐H^+^⋅BB1 **1‐H^+^** ⋅ **3** heterodimers. The observed proton dependent behavior in the system is thus fully consistent with the binding behavior and complexation affinities.

**Figure 3:**
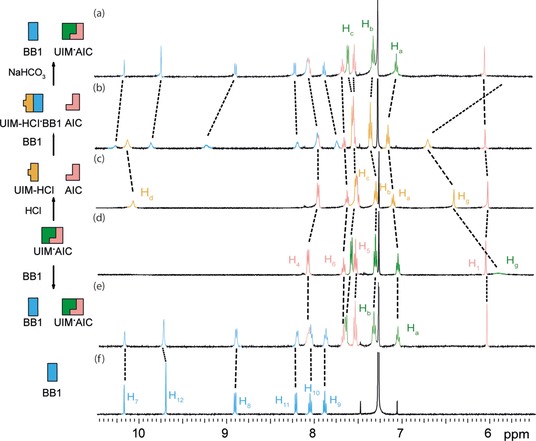
Self‐sorting behavior of proton responsive UIM **1** (condition A) with AIC **2** and BB1 **3** studied by ^1^H NMR (500 MHz, 5 mM, CDCl_3_) (a) UIM⋅AIC **1** ⋅ **3** and BB1 **3** formed after washing UIM‐HCl⋅BB1 **1‐H^+^** ⋅ **3** and AIC **2** mixture with NaHCO_3_ (b) UIM‐HCl⋅BB1 **1‐H^+^** ⋅ **3** (protonated with 4 M HCl in 1,4‐dioxane) and AIC **2**, (c) UIM‐HCl **1‐H^+^** (protonated with 4 M HCl in 1,4‐dioxane) and AIC **2**, (d) UIM⋅AIC **1** ⋅ **2**, (e) UIM⋅AIC **1** ⋅ **3** and BB1 **3** and (f) BB1 **3**. Refer to Figure 1 for the resonance assignment and chemical structure.

**Table 1:** Association constants (determined by ^1^H NMR titration, 500 MHz, CDCl_3_) for complexes involving UIM **1**, AIC **2**, BB1 **3** and UIM‐H^+^ **1‐H^+^**.

Complex	*K*_a_ (M^−1^)
UIM⋅AIC **1** ⋅ **2**	3.3×10^4^ (±0.8×10^4^)
UIM⋅BB1 **1** ⋅ **3**	2×10^3^
AIC⋅BB1 **2** ⋅ **3**	No CIS at 10 μM
UIM‐H^+^⋅BB1 **1‐H^+^** ⋅ **3**	≥1×10^5^
UIM‐H^+^⋅AIC **1‐H^+^** ⋅ **2**	No CIS at 10 μM

In the studies described above, the chloride ion used in generating **1‐H^+^** may compete for the hydrogen‐bond donor groups, complicating further the equilibria and thus spectral interpretation. Consequently, the chloride ion of **1‐H^+^** was exchanged for a non‐competitive anion (hexafluorophosphate (PF_6_)) and the molecular recognition of UIM‐HPF_6_ with complementary and competing HBMs (**2** and **3**) was examined (see Scheme ESI 1 and Figure ESI 10 & 11). Overall the recognition behaviour exhibited by UIM‐HPF_6_ closely mimics the preferences exhibited by HBM UIM‐HCl **1‐H^+^** (full discussion can be found in the supporting information). Finally, it should be noted that there is potential for AIC **2** and BB1 **3** to become protonated; indeed spectral changes are observed upon addition of HCl to either component (see Figure ESI 12 for details), however for AIC **2**, in the presence of other components i. e. UIM **1**, AIC **2** is not protonated whilst for BB1 **3**, it is more instructive to consider the **1** ⋅ **3** complex as the preferred site of protonation.

In summary we have used acid‐base chemistry to modulate the recognition preference of a HBM to demonstrate that UIM⋅AIC **1** ⋅ **2** and UIM‐H^+^⋅BB1 **1‐H^+^** ⋅ **3** heterodimers are the preferred complexes in three component mixtures. This stimuli dependent behavior is distinct from prior studies on stimuli dependent H‐bonding^30^, ^31^, ^32^, ^33^, ^34^, ^35^, ^36^, ^37^ which have centered on switching recognition on/off or potentiating affinity. Crucially, the results illustrate that switching of molecular recognition preference for HBMs is feasible in a simple self‐sorting network. Future efforts will focus on optimizing the design and reagents to permit multiple cycles of switching between the two self‐sorted states, exploring the incorporation of this and other responsive hydrogen motifs in more complex self‐sorting networks and development of functional biomimetic self‐sorting networks.

## Experimental Section

See supporting information for full experimental details.

## Supporting information

As a service to our authors and readers, this journal provides supporting information supplied by the authors. Such materials are peer reviewed and may be re‐organized for online delivery, but are not copy‐edited or typeset. Technical support issues arising from supporting information (other than missing files) should be addressed to the authors.

Supplementary
