# Supplementary material for: A pH‐Switchable Triple Hydrogen‐Bonding Motif
Source: ChemistryOpen. 2020 Jan 8;9(1):40–4. doi: 10.1002/open.201900338 (PMC6948117; doi:10.1002/open.201900338)
Supplement: Supplementary file 1 — Supplementary [file OPEN-9-40-s001.pdf]

Open Access
ChemistryOPEN

Supporting Information

© Copyright Wiley-VCH Verlag GmbH & Co. KGaA, 69451 Weinheim, 2020

A pH-Switchable Triple Hydrogen-Bonding Motif

Heather M. Coubrough, Barbora Balonova, Christopher M. Pask, Barry A. Blight, and Andrew J. Wilson*© 2020 The Authors. Published by Wiley-VCH Verlag GmbH & Co. KGaA. This is an open access article under the terms of the Creative Commons Attribution License, which permits use, distribution and reproduction in any medium, provided the original work is properly cited.An invited contribution to a Special Collection dedicated to Functional Supramolecular Systems

Contents

1. ESI Figures of additional NMR studies. 2

Data for Condition A 2

Data for Condition B 3

Studies with Hexafluorophosphate Anion 9

NMR Titration Experiments 5

2. ESI Schemes of synthesis 9

3. General materials and methods for synthesis 14

3, 5-diiodo-2,6-diaminopyridineamine 14

Benzoisoquinolino-napthypyridine BB1 (3) 15

4-tert-Butyl-1*H*-imidazole-2-amine hydrochloride (*I*) 15

4. General procedure for preparation of 5-*tert*-Butyl-2-[{phenylcarbamoyl}amino]-1*H*-imidazol-3-ium acid salts 17

Hydrochloride salt (1-H^+) (5-*tert*-Butyl-2-[{phenylcarbamoyl}amino]-1*H*-imidazol-3-ium chloride) 17

Trifluoroacetate salt (1-H^+) (5-*tert*-Butyl-2-[{phenylcarbamoyl}amino]-1*H*-imidazol-3-ium trifluoroacetate) 17

Hexafluorophosphate salt (1-H^+) (5-*tert*-Butyl-2-[{phenylcarbamoyl}amino]-1*H*-imidazol-3-ium hexafluorophosphate) 17

5. General procedure for sample preparation for NMR switching experiments 18

Condition A 18

Condition B 18

6. Spectral Data 19

7. References 23

1. ESI Figures of additional NMR studies.

Data for Condition A

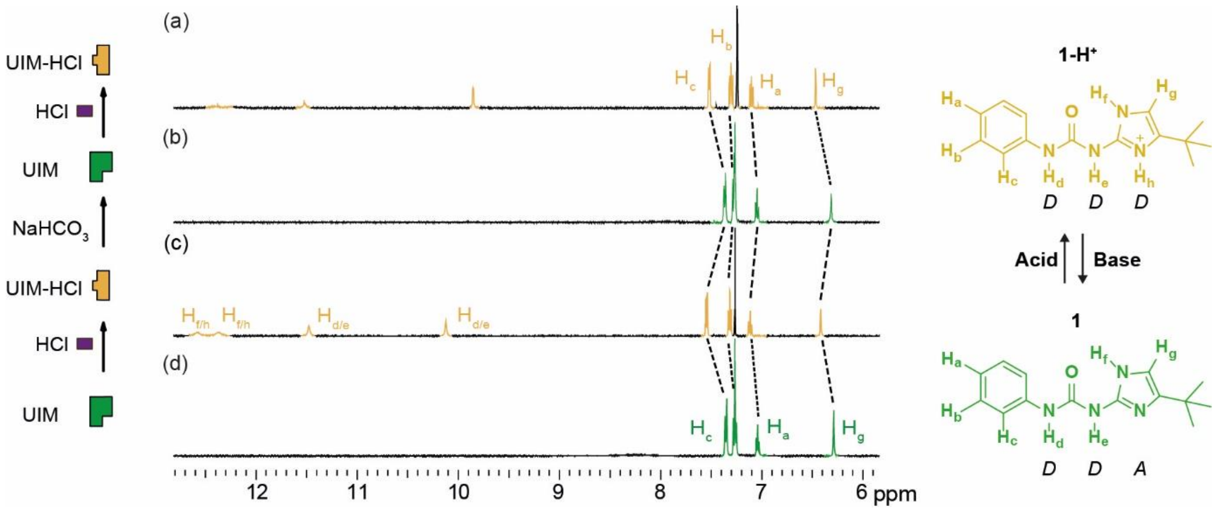

Figure ESI 1. Analysis of UIM (de)protonation by ^1H NMR (500 MHz, 10 mM, $CDCl_3$), (a) UIM-HCl **1·H⁺** (protonated with 4M HCl in 1,4-dioxane cycle 2), (b) UIM **1** (deprotonated with $NaHCO_3$ cycle 1), (c) UIM-HCl **1·H⁺** (protonated with 4M HCl in 1,4-dioxane cycle 1), (d) UIM **1**.

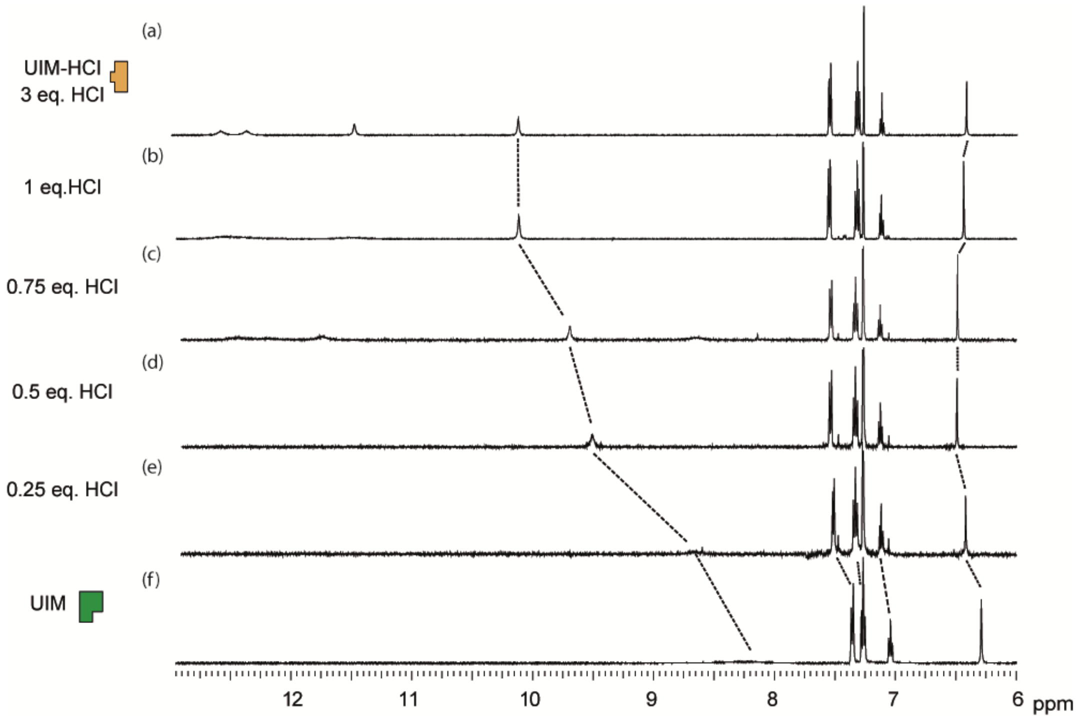

Figure ESI 2. Titration of HCl into UIM **1** studied by ^1H NMR (500 MHz, 5 mM, $CDCl_3$) (a) UIM **1** with 3 eq. HCl, (b) UIM **1** with 1 eq. HCl, (c) UIM **1** with 0.75 eq. HCl, (d) UIM **1** with 0.5 eq. HCl, (e) UIM **1** with 0.25 eq. HCl and (f) UIM **1**.

Data for Condition B

The use of condition B: trifluoroacetic acid (TFA) and 1,4-diazabicyclo[2.2.2]octane (DABCO), to protonate **1** and deprotonate **1-H**$^+$ HBM was studied. ^{1}H NMR analysis revealed a downfield shift of diagnostic resonances, H_a, H_b, H_c and H_g. on the addition of 1 equivalent of TFA to UIM **1** (5 mM in $CDCl_3$) as observed for condition A (Figure ESI 3 (d)-(c)). This suggests TFA is also able to protonate UIM **1** to form UIM-TFA **1-H**$^+$. To reverse the protonation, one equivalent of DABCO was added to the 5 mM solution of UIM-TFA **1-H**$^+$ (Figure ESI 3 (c)-(b)). This resulted in small changes in the chemical shifts of the diagnostic proton resonances H_a, H_b, H_c and H_g but the resonances did not fully align with those observed for neutral UIM **1** (or a 1:1 UIM:DABCO mixture) suggesting a mixture of neutral and protonated UIM. However, with addition of excess DABCO (3 eq.) the diagnostic protons shifted further upfield consistent with formation of UIM **1** as the dominant species (Figure ESI 3 (a)). The lower pK_a of DABCO in comparison to sodium hydrogen carbonate dictate that a greater concentration of DABCO for full deprotonation is to be expected. Similarly the pKa of DABCO is close to imidazolium (which forms the core of UIM).

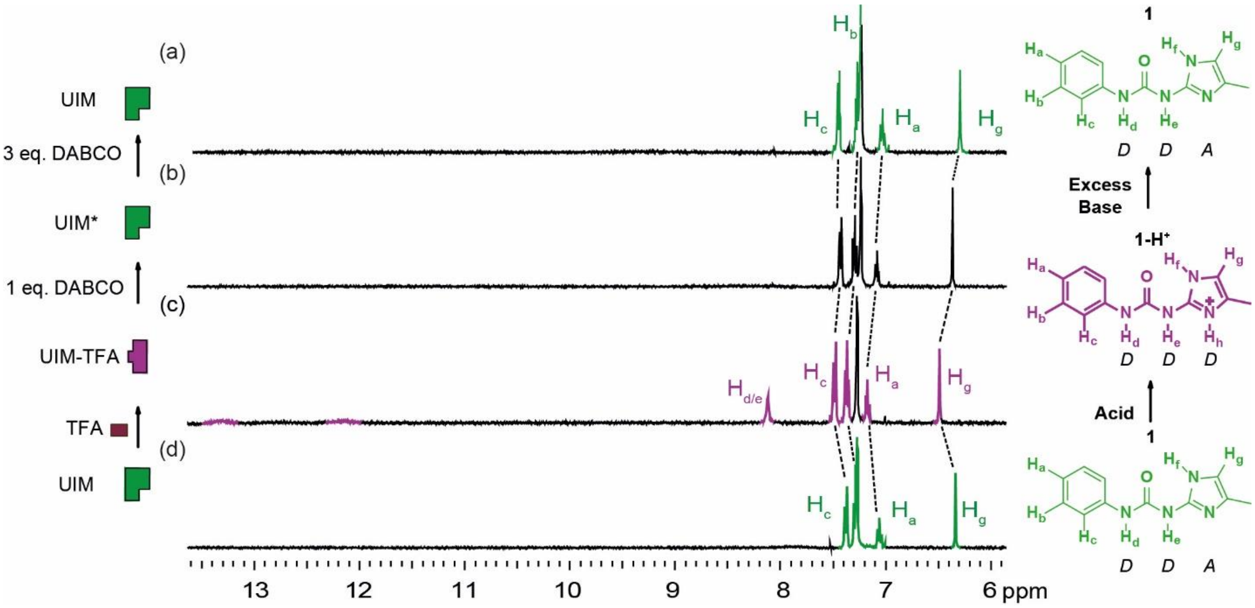

Figure ESI 3. Condition B proton switch studied by ^{1}H NMR (500 MHz, 10 mM, $CDCl_3$) (a) UIM **1** (deprotonated with 3 eq DABCO), (b) UIM **1** (deprotonated with 1 eq DABCO), (c) UIM-TFA **1-H**$^+$ (protonated with TFA), (d) UIM **1**.

The switching 'off' behaviour of the **1·2** dimer interaction was tested using condition B. On the addition of 1 equivalent of TFA to the **1·2** dimer the diagnostic H_g resonance moved significantly downfield and became well resolved, unlike in the **1·2** dimer (Figure ESI 4 (c)-(b)). Additionally the presence of NH resonance (H_d) of UIM, not seen in neutral UIM **1** or UIM·AIC **1·2** dimer, indicated protonation of UIM **1** to give UIM-TFA **1-H**$^+$. The addition of DABCO to this mixture indicated that the *ADD-DAA* dimer was reformed by the upfield shift and broadening of the H_g resonance (Figure ESI 4 (a). However, some of the resonances associated with **1** (H_a and H_c) did not fully match in the **1·2** dimer as anticipated. This could be a result of partial deprotonation using DABCO, compared to sodium hydrogen carbonate (used in condition A), leading to a mixture of species as well as TFA and DABCO salts.

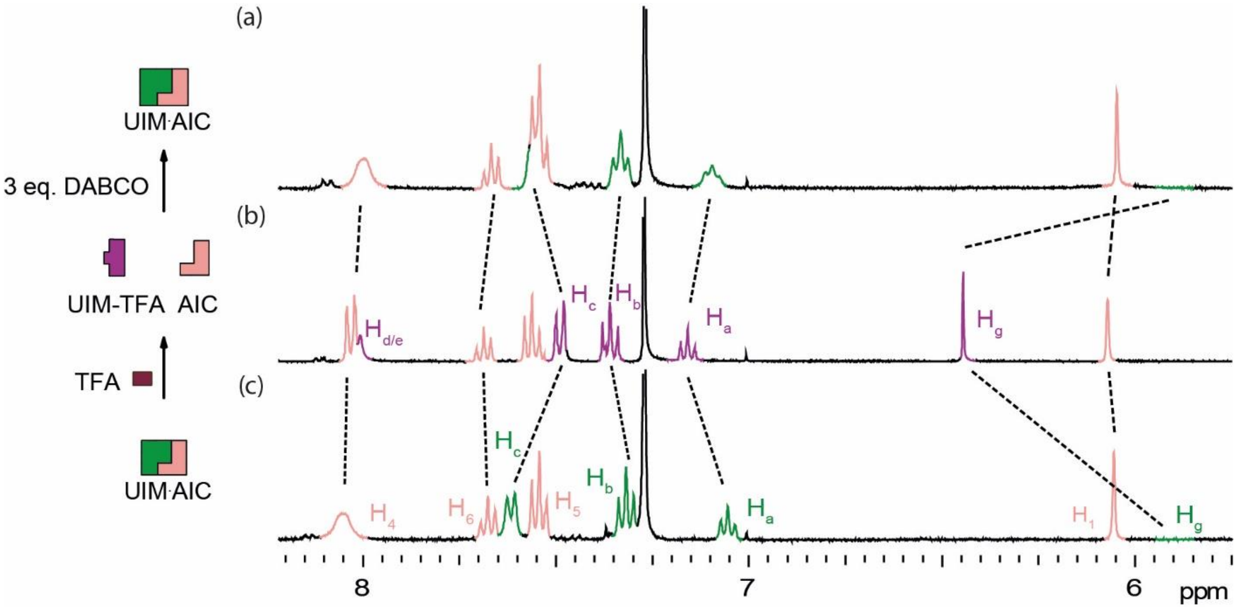

Figure ESI 4. Self-sorting behaviour of proton responsive UIM **1** (condition B) with AIC **3** studied by ^{1}H NMR (500 MHz, 5 mM, $CDCl_3$) (a) UIM·AIC **1·2** (dimer formed after washing UIM-TFA **1-H$^+$** and AIC **3** mixture with 3 eq. DABCO), (b) mixture of UIM-TFA **1-H$^+$** and AIC **2** (formed after protonation with TFA) and (c) UIM·AIC **1·2** dimer. Refer to Figure ESI 3 for the resonance assignment and chemical structure.

The recognition preferences of complementary and competing HBMs, **2** and **3**, with protonated and neutral HBM **1** formed using condition B were studied. Addition of TFA to a mixture of the UIM·AIC **1·2** dimer and HBM BB1 **3** disrupted the UIM·AIC **1·2** dimer generating a mixture of UIM-TFA·BB1 **1-H$^+$·3** dimer in presence of AIC **2** (Figure ESI 5 (b)). This switch is highlighted by a change in UIM resonances; the sharpening and downfield shift of diagnostic H_g resonance and the presence of NH resonance H_d. As well as more subtle chemical shifts in the resonances of AIC **2** and BB1 **3**. The addition of 3 equivalents of DABCO to this mixture did not fully switch back ‘on’ UIM·AIC **1·2** dimerization (Figure ESI 5 (a)); although some shifts in the spectra are observed these do not return to those observed for the spectrum of UIM·AIC **1·2** dimer and HBM BB1 **3** (Figure ESI 5 (c)) before the protonation/deprotonation cycle. This likely results from greater competition between DABCO and the **1·3** complex for the proton i.e. a reduced difference in pK_a between the two. Aside from the diminished reversibility, the interactional preferences for conditions B are in line with the observations for conditions A.

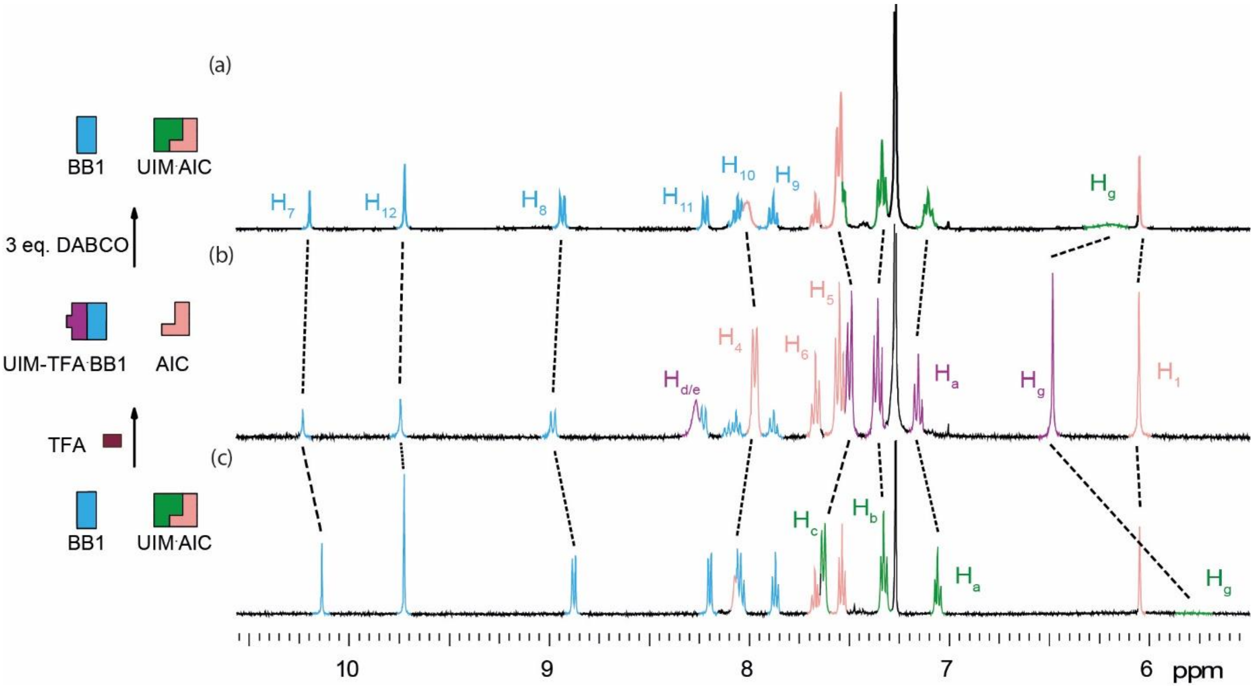

Figure ESI 5. Self-sorting behaviour of proton responsive UIM **1** (condition B) with AIC **2** and BB1 **3** studied by ^{1}H NMR (500 MHz, 5 mM, $CDCl_3$) (a) UIM·AIC **1·2** dimer and BB1 **3** formed after washing UIM-HCl·BB1 **1-H⁺·3** dimer and AIC **2** mixture with 3 eq. DABCO, (b) UIM-TFA·BB1 **1-H⁺·3** dimer (protonated with TFA) and AIC **2** and (c) UIM·AIC **1·2** dimer and BB1 **3**. Refer to Figure ESI 3 for the resonance assignment and chemical structure.

NMR Titration Experiments

For ^{1}H NMR titrations anhydrous $CDCl_3$ was purchased from Aldrich and stored over molecular sieves (type 4A, 1 to 2mm beads). For the qualitative titration study of hydrochloric acid into UIM **1** the ^{1}H NMR spectrum was recorded for a solution of host (5 mM) in $CDCl_3$ and the change in chemical shift of key proton resonances was recorded upon sequential additions of a solution of guest (0.25-3 equivalents) in $CDCl_3$. For the quantitative titration study of UIM **1** and UIM-HCl **1-H⁺** into BB1 **3**, the ^{1}H NMR spectrum was recorded for a solution of host (0.5 mM) in $CDCl_3$ and the change in chemical shift of key proton resonances was recorded upon sequential additions of a solution of guest (0.2-12.3 equivalents) in $CDCl_3$. The solutions of guests (UIM **1** and UIM-HCl **1-H⁺**) were made through a half dilution series and added to a solution of host (BB1 **3**). The equivalents were calculated by the ratio of integration of individual resonances. The data was subsequently analysed using the Supramolecular.org online bindfit program using the appropriate model to give an association constant.[1,2] Supramolecular.org uses data from multiple resonances for curve fitting. Representative ^{1}H NMR spectra and exported binding curves can be seen below.

Titration of UIM **1** into BB1 **3**

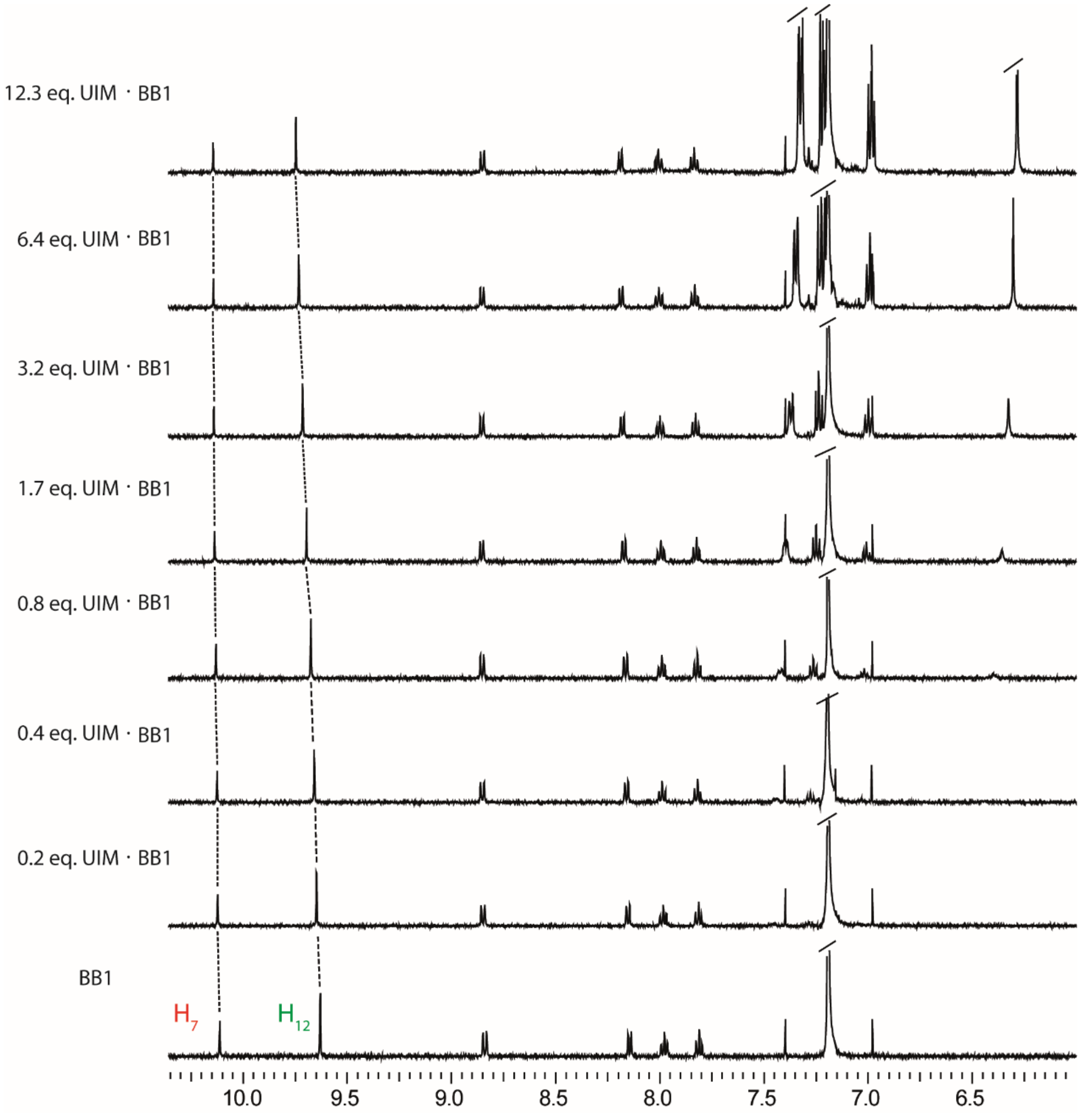

Figure ESI 6. Titration of UIM **1** (0.2-12.3 eq.) into BB1 **3** (0.5 mM) studied by ^{1}H NMR (500 MHz, $CDCl_3$). Refer to Figure 1 for the resonance assignment and chemical structure.

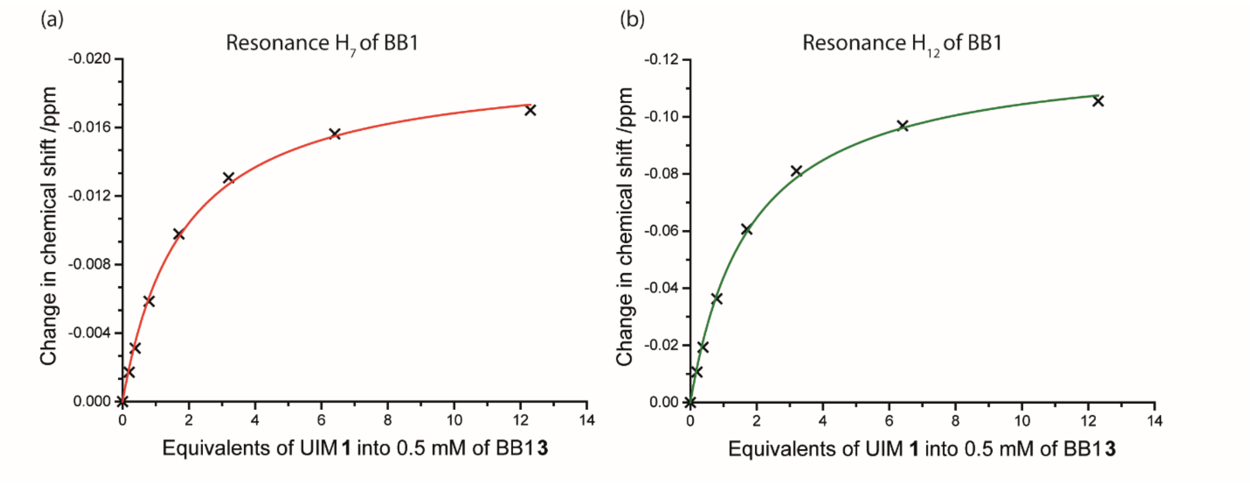

Figure ESI 7. Supramolecular.org fitting of the titration of UIM **1** (0.2-12.3 eq.) into BB1 **3** (0.5 mM) using the chemical shift of BB1 **3** (a) resonance H_7 and (b) resonance H_{12}.

Titration of UIM-HCl **1-H⁺** into BB1 **3**

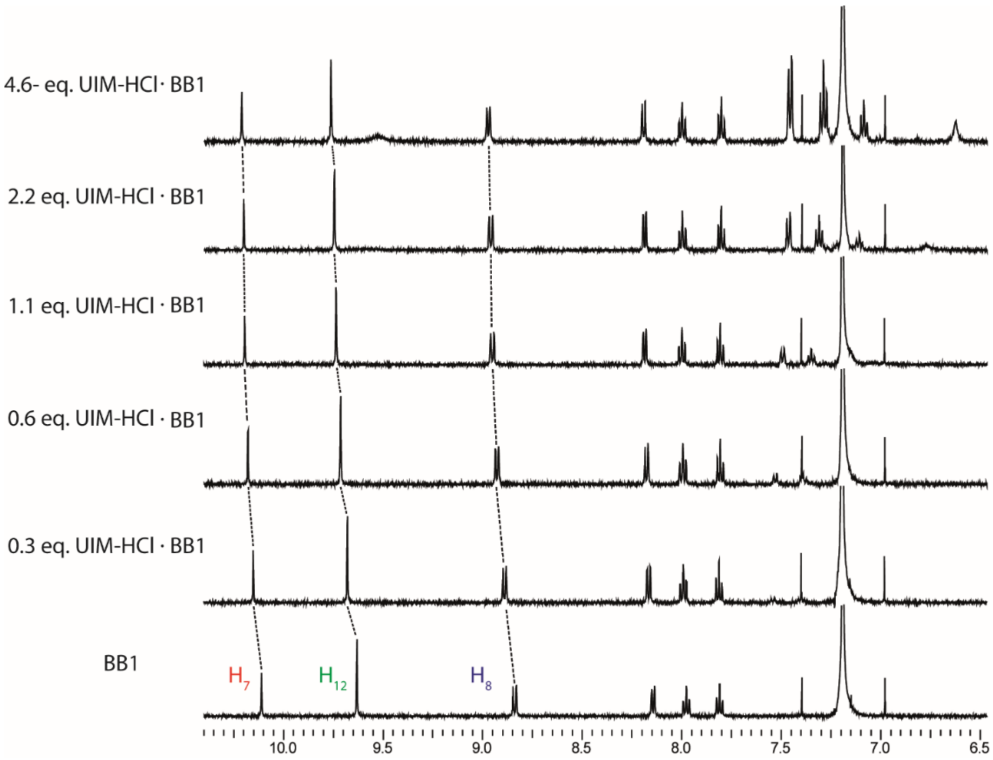

Figure ESI 8. Titration of UIM **1-H$^+$** (0.3-4.6 eq.) into BB1 **3** (0.5 mM) studied by ^{1}H NMR (500 MHz, $CDCl_3$). Refer to Figure 1 for the resonance assignment and chemical structure.

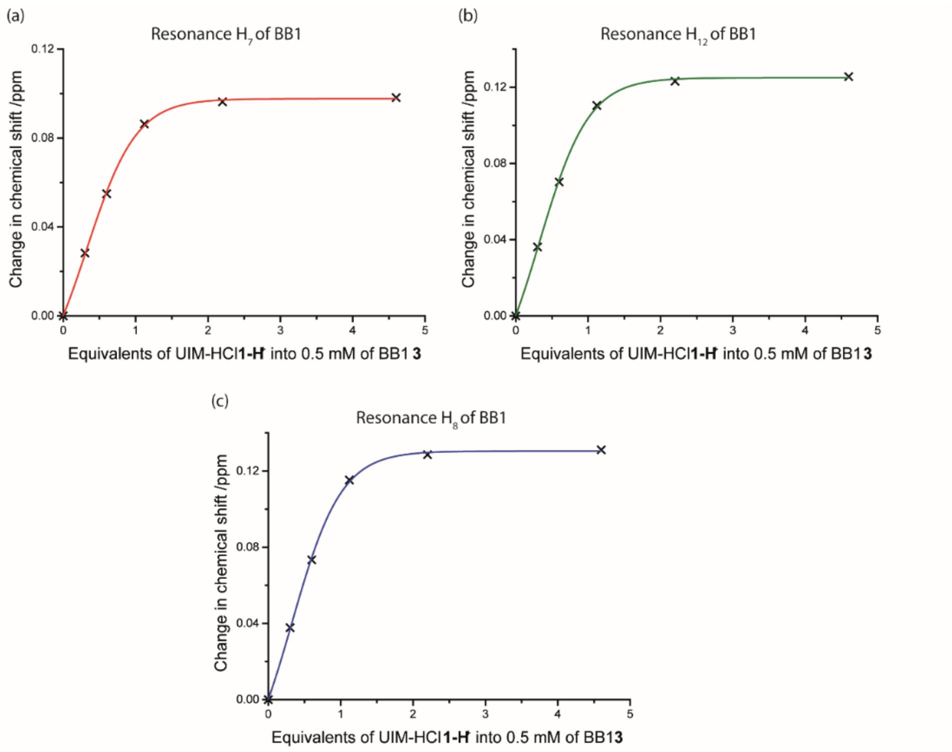

Figure ESI 9. Supramolecular.org fitting of the titration of UIM **1-H**$^+$ (0.3-4.6 eq.) into BB1 **3** (0.5 mM) using the chemical shift of BB1 **3** (a) resonance H_7, (b) resonance H_{12} and (c) resonance H_8.

Studies with Hexafluorophosphate Anion

Reflecting on the crystal structure of the intermediate ***I*** (Figure 1(a)) where the chloride ion bridges two NH groups, the chloride ion may influence the equilibria (Scheme ESI 1); although the time averaged nature of the ^{1}H NMR analysis reports provides information that can be related to the interactions of the HBMs, the coordination of chloride ion could alter the desired hydrogen bonding interactions between the HBMs.

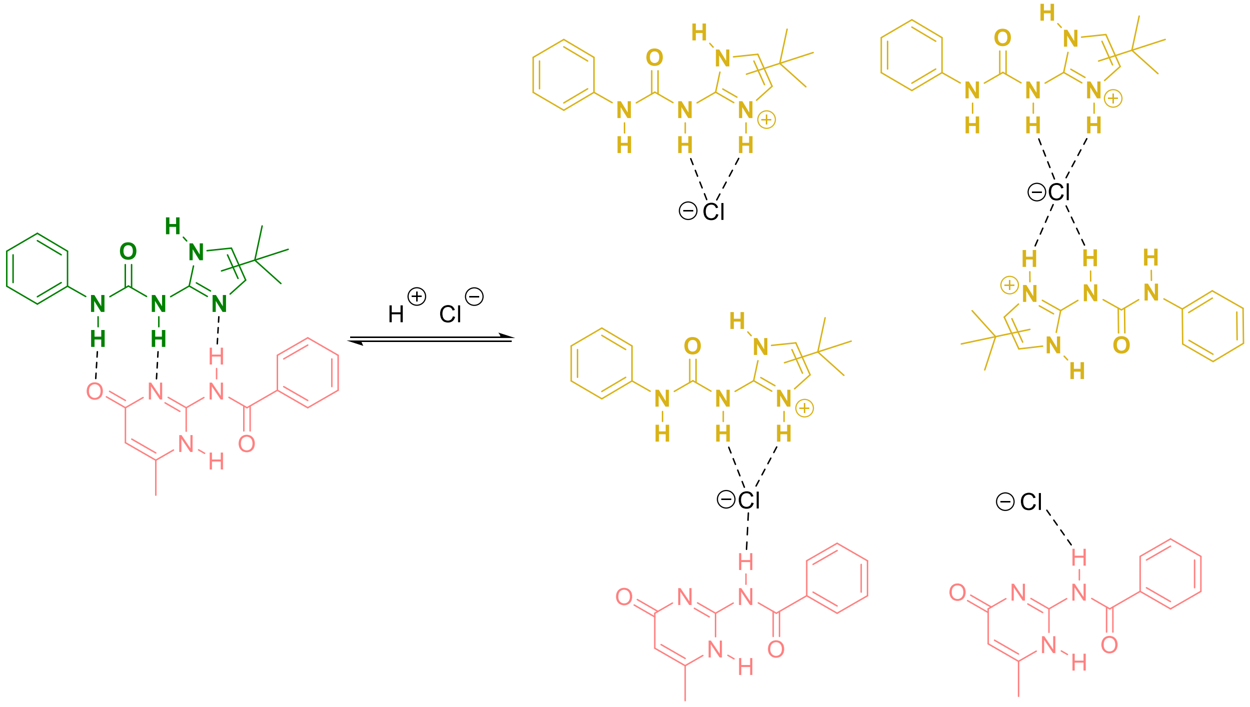

Scheme ESI 1. Schematic UIM-HCl **1-H**$^{+}$ showing potential interactions of chloride ion with the components UIM-HCl **1-H**$^{+}$ and AIC **2**.

To assess this, the chloride ion was exchanged for a non-interacting anion, hexafluorophosphate (PF_6) using silver hexafluorophosphate to create UIM-HPF$_6$ **1-H**$^{+}$PF$_6$ (Figure ESI 10). ^{1}H NMR suggested that after the addition of silver hexafluorophosphate the species was still protonated; as the chemical shift of the imidazole CH resonance (H_g) was similar to that observed for UIM-HCl **1-H**$^{+}$ and the NH resonances (H_d, H_e, H_f and H_h) were well-resolved (Figure ESI 10 (b)). However, the NH resonances (H_d, H_e, H_f and H_h) shifted significantly for UIM-HPF$_6$ **1-H**$^{+}$PF$_6$ compared to the resonances observed for UIM-HCl **1-H**$^{+}$. Next, proton dependent switching of UIM-HPF$_6$ **1-H**$^{+}$PF$_6$ was explored, revealing it was possible to deprotonate motif UIM-HPF$_6$ **1-H**$^{+}$PF$_6$ by washing with $NaHCO_3$ in the same way that UIM-HCl **1-H**$^{+}$ was deprotonated using condition A (Figure ESI 10 (a)).

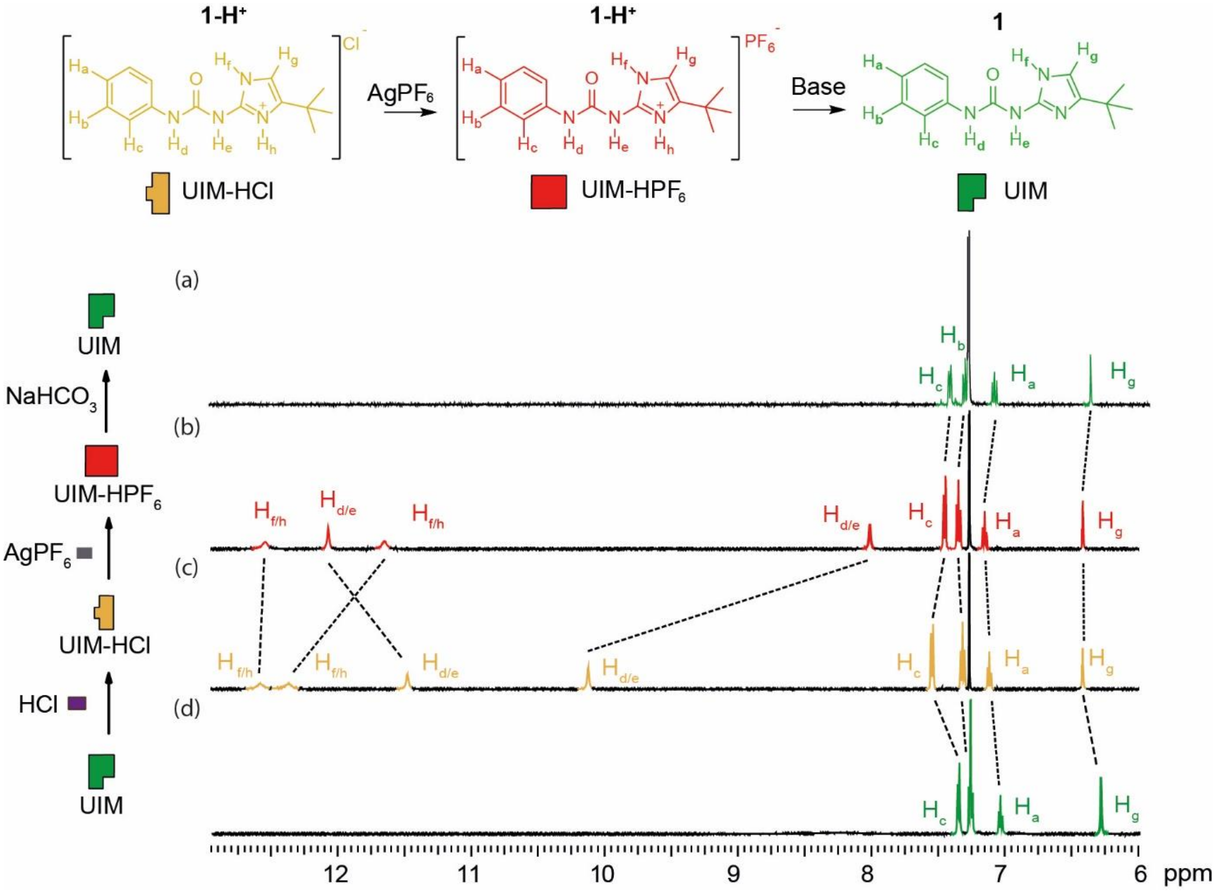

Figure ESI 10. Formation of non-interacting motif UIM-HPF$_6$ **1-H**$^+$PF$_6$ and subsequent deprotonation to form UIM **1** studied by ^{1}H NMR (500 MHz, 5 mM, CDCl$_3$) (a) UIM **1** (deprotonated UIM-HPF$_6$ with NaHCO$_3$), (b) UIM-HPF$_6$ **1-H**$^+$PF$_6$ (formed on the addition of AgPF$_6$ to UIM-HCl **1-H**$^+$), (c) UIM-HCl **1-H**$^+$ (protonated with 4M HCl in 1,4-dioxane) and (d) UIM **1**.

The self-sorting preference of UIM-HPF$_6$ **1-H**$^+$PF$_6$ compared to UIM-HCl **1-H**$^+$ was investigated by ^{1}H NMR upon addition of HBMs AIC **2** and BB1 **3** (Figure ESI 11). As expected UIM-HPF$_6$ **1-H**$^+$PF$_6$ was able to interact with complementary BB1 **3** (Figure ESI 11 (c)-(d)) and no interaction was seen on the addition of AIC **2** (Figure ESI 11 (c)-(b)), matching the behaviour observed for UIM-HCl **1-H**$^+$. Note: the NH resonances of UIM-HPF$_6$ **1-H**$^+$PF$_6$ are broadened on the addition of AIC **2** (Figure ESI 6 (b)), in comparison to UIM-HPF$_6$ **1-H**$^+$PF$_6$ alone Figure ESI 6c) which may arise as a consequence of proton exchange.

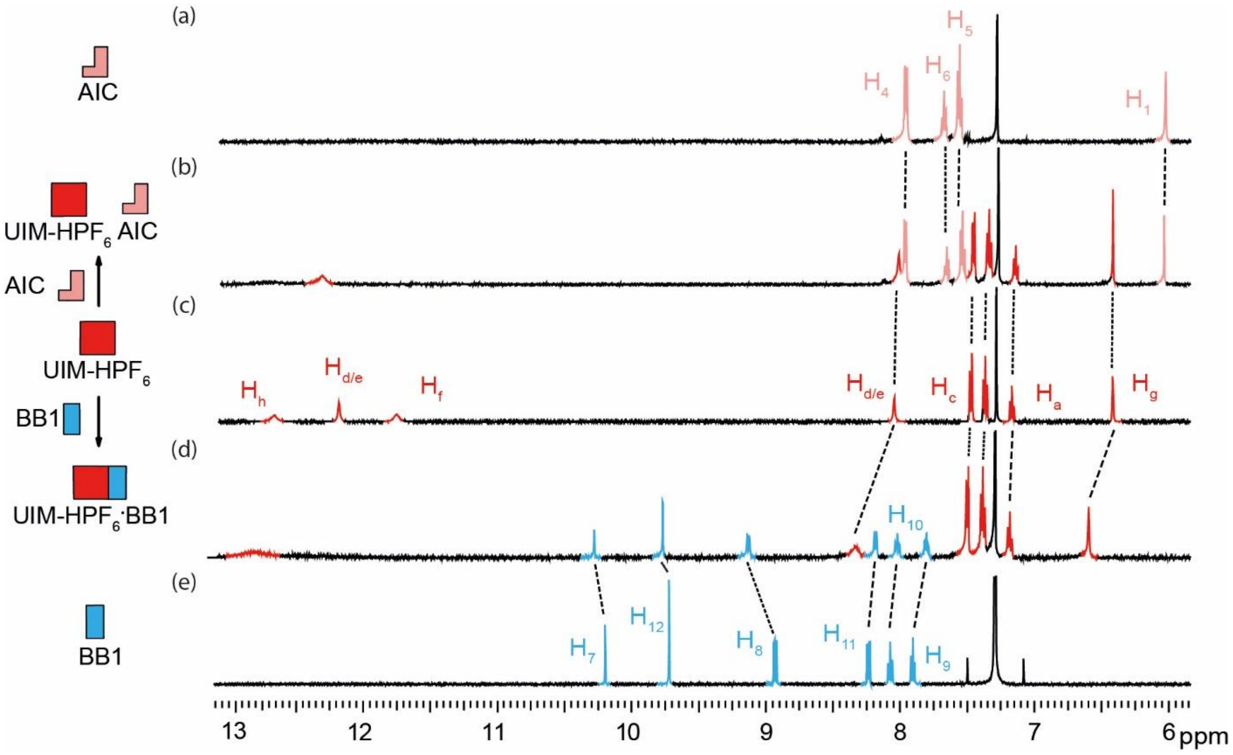

Figure ESI 11. Recognition behavior of proton responsive UIM-HPF$_6$ **1-H**$^+$PF$_6$ with AIC **2** and BB1 **3** studied separately by ^{1}H NMR (500 MHz, 5 mM, CDCl$_3$) (a) AIC **2**, (b) UIM-HPF$_6$ **1-H**$^+$PF$_6$ and AIC **2**, (c) UIM-HPF$_6$ **1-H**$^+$PF$_6$, (d) UIM-HPF$_6$·BB1 **1-H**$^+$PF$_6$ **·3** dimer and (e) BB1 **3**. Refer to Figure ESI 10 for the resonance assignment and chemical structure

When AIC **2** and BB1 **3** were combined with UIM-HPF$_6$ **1-H**$^+$PF$_6$, the ^{1}H NMR spectrum was indicative of an interaction between BB1 **3** and UIM-HPF$_6$ **1-H**$^+$PF$_6$, while the chemical shifts of AIC **2** were unchanged in comparison to the isolated samples (Figure ESI 4(c)-(b)). Efforts were made to switch this self-sorting behaviour 'off' by the addition of base to reform the UIM·AIC **1·2** dimer in the presence of BB1 **3**. However due to the lower solubility of BB1 **3** and the process of washing with NaHCO$_3$, sample was lost, thus it was not possible to obtain a well resolved ^{1}H NMR spectra with equal ratios of hydrogen bonding motifs. The limited solubility of BB1 **3** in CDCl$_3$ was more profound in the system using PF$_6$ salts than the hydrochloric acid containing systems. Overall the recognition behaviour exhibited by UIM-HPF$_6$ **1-H**$^+$PF$_6$ closely resembled the preferences exhibited by HBM UIM-HCl **1-H**$^+$ indicating that whilst the counter anion may influence the equilibria under analysis, the ^{1}H NMR data reflect accurately on the recognition preference of the HBMs in these mixtures.

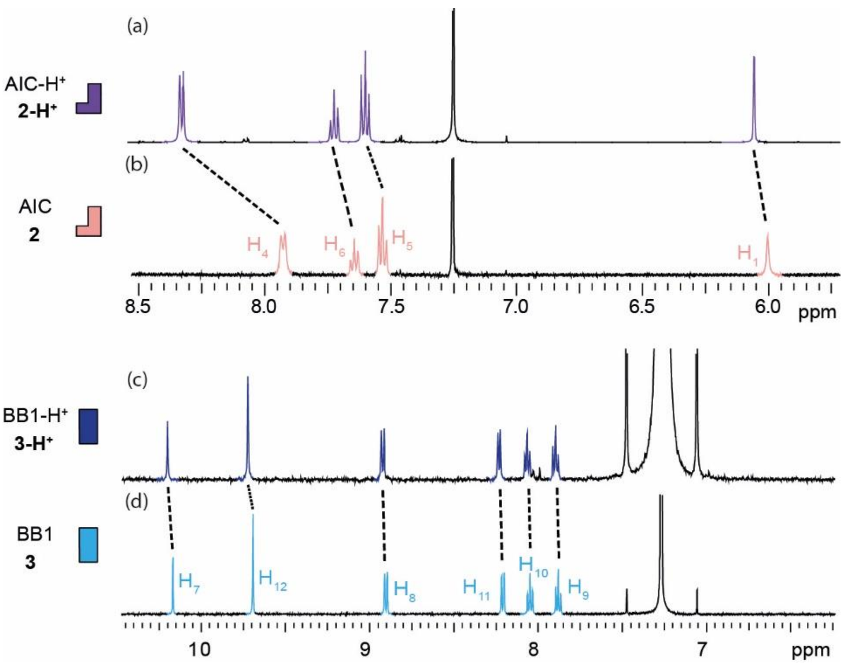

Figure ESI 12. Protonation behaviour of AIC **2** and BB1 **3** studied by ^{1}H NMR (500 MHz, $CDCl_3$) (a) protonated AIC with HCl **2-H^{+},** (b) AIC **2,** (c) protonated BB1 with HCl **3-H^{+}**and (d) BB1 **3**. Refer to Figure 1 for the resonance assignment and chemical structure.

2. ESI Schemes of synthesis

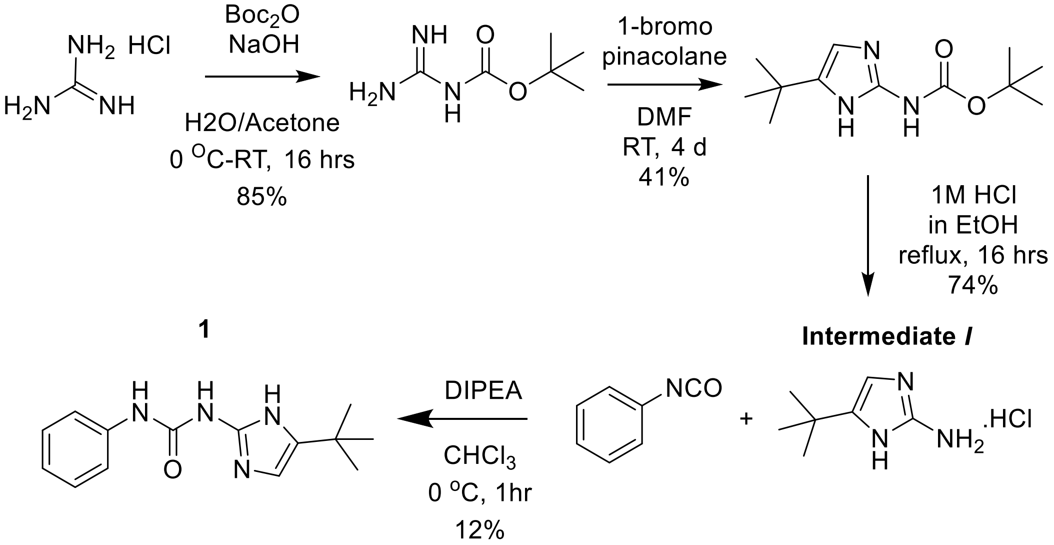

Scheme ESI 2. The synthetic route for the preparation of UIM **1**.[3]

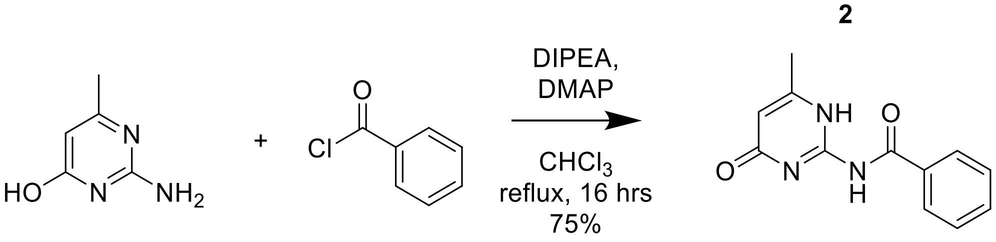

Scheme ESI 3. The synthetic route for the preparation of AIC **2**.[3]

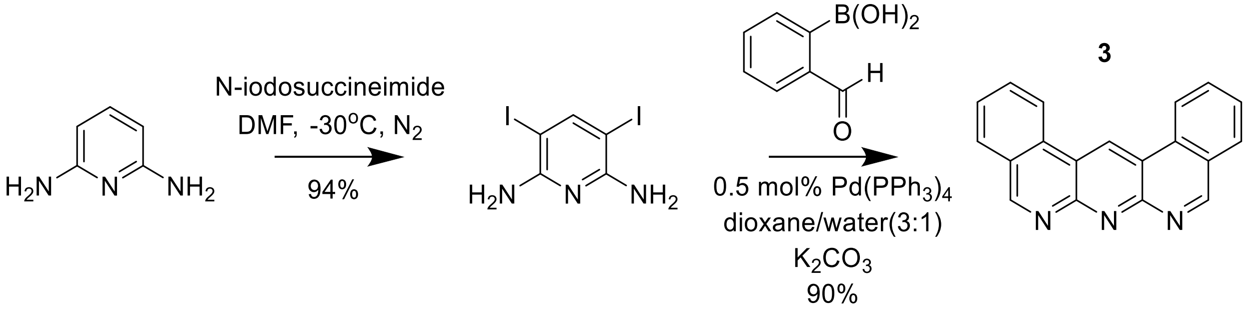

Scheme ESI4. The synthetic route for the preparation of BB1 **3**.[4,5]

3. General materials and methods for synthesis

Solvents and reagents were purchased from Sigma Aldrich or Fisher Scientific and used without further purification unless otherwise stated. Where anhydrous solvents were required, dichloromethane, chloroform, tetrahydrofuran and acetonitrile were obtained from the in-house solvent purification system Innovative Inc. PureSolv®. Anhydrous dimethyl formamide and *N*,*N*-diisopropylethylamine were obtained from Sigma Aldrich equipped with Sure/Seal™. All non-aqueous reactions were carried out under a nitrogen atmosphere. Chloroform-d was dried over Linde 5Å molecular sieves or placed on $CaCl_2$ before being distilled and stored on KOH prior to use in ^{1}H NMR experiments. For reactions under non-anhydrous conditions, the solvents used were HPLC quality and provided by Sigma Aldrich or Fisher. Water in aqueous solutions and used for quenching was deionised. Mixtures of solvents are quoted as ratios and correspond to a volume: volume ratio. Analytical thin layer chromatography was performed on Merck Kieselgel 60 F_{254} 0.25 mm pre-coated aluminium plates. Product spots were visualised under UV light (λmax = 254 nm) or using a suitable stain. Flash chromatography was carried out using Merck Kieselgel 60 silica gel using pressure by means of head bellows or using disposable RediSepRf silica flash columns on an automated Biotage Isolera One system. Nuclear magnetic resonance spectra were obtained at 298 K (unless stated) using a Bruker AV500 spectrometer operating at 11.4 T (500 MHz for ^{1}H and 125 MHz ^{13}C) and JEOL ECA600ii operating at 14.1 T (150 MHz for ^{13}C) and NOESY spectra as stated. Infra-red spectra were obtained using a Bruker Alpha Platinum ATR where absorption maxima (ν_{max}) are quoted in wavenumbers (cm^{-1}) and only structurally relevant absorptions have been included. High Resolution Mass Spectra (HRMS) were recorded on a Bruker Daltonics Micro TOF using electrospray ionisation (ESI). Liquid Chromatography and Mass Spectrometry (LC-MS) was performed using an Agilent Technologies 1200 series LC and a Bruker HCT ultra ion-trap MS.

Compounds **1** (UIM)] and **2** (AIC) were synthesized as described previously.[3] Compound **3** was synthesised as below.

3, 5-diiodo-2,6-diaminopyridineamine

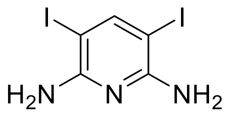

Compound prepared using minor adaptations to a previously described procedure.[4] To a solution of 2,6-diaminopyridine (0.99 g, 9.09 mmol) in dry DMF (28 mL) *N*-iodosuccineimide (4.50 g, 19.9 mmol) in DMF (20 mL) was added dropwise at -30 °C (cooled with dry ice) over 1 hr. After the addition was completed, the cooling bath was removed and the reaction mixture was stirred for 1 hr and then poured into ice-cold water and stirred for 30 min. Resulting precipitate was filtered and washed with water (2 x 20 mL) and pentane (2 x 15 mL) and dried in vacuum oven at 40 °C for 24 hrs. Product was obtained as grey solid with 94 % yield. ^{1}H-NMR (500 MHz, $CDCl_3$): δ ^{1}H NMR (400 MHz, DMSO-d_6) δ: 7.73(s, 1H, Ar-*H*), 5.71(s, 4H, N*H*$_2$). ^{13}C NMR (100 MHz, DMSO-d_6) δ: 157.06, 153.81, 60.18. Characterization in accordance with literature.

Benzoisoquinolino-napthypyridine BB1 (3)

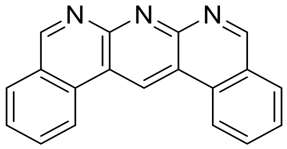

Compound prepared using minor adaptations to a previously described procedure.[5] The mixture of 2-formylphenylboronic acid (0.36g, 2.42 mmol), 3, 5-diiodo-2,6-pyridinediamine (0.43g, 1.21 mmol), potassium carbonate (12 equiv.) and tetrakis triphenylphosphine palladium (5 mol%, ~20mg) with dioxane and water (3:1) was heated in microwave at 150 °C for 15 mins. After the reaction was completed, the mixture was cooled down to r.t. and stirred for 30 mins, the product crushed out from the solution. Resulting precipitate was filtered, washed with water (3 x 10 mL) and dried in vacuum oven at 40°C for 24h. Title compound **BB1** was obtained in 90% yield as yellowish compound. Characterization in accordance with literature.

4-tert-Butyl-1*H*-imidazole-2-amine hydrochloride (*I*)

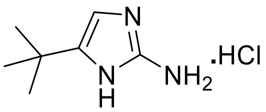

Tert-butyl 5-*tert*-1H-imidazol-2-yl-carbamate (750 mg, 3.10 mmol) was dissolved in 1M HCl in ethanol (30 mL) and refluxed for 16 hrs. The reaction was allowed to cool, concentrated and dried under pressure to give a colourless solid (400 mg, 2.28 mmol, 74%) ^{1}H-NMR (500 MHz, $CDCl_3$): δ 11.83 (s, 1H, N*H*), 11.23 (s, 1H, N*H*). 6.24 (s, 1H, imidazole-*H*), 5.53 (br s, 2H, NH_2), 1.25 (s, 9H, CH_3 (C^tBu)) ppm; ^{13}C-NMR (125 MHz, DMSO-d_6): δ 147.1, 135.8, 106.1, 29.8, 28.6 ppm; ν_{max} (solid state) = 3500-2800 (broad) cm^{-1}; ESI-HRMS *m/z* found 140.1190 $[M + H]^+$ $C_7H_{14}N_3$ requires140.1188.

X-ray Crystallography of Intermediate ***I***.

Measurements were carried out at 120K on an Agilent SuperNova diffractometer equipped with an Atlas CCD detector and connected to an Oxford Cryostream low temperature device using mirror monochromated Cu K_α radiation (λ = 1.54184 Å) from a Microfocus X-ray source. The structure was solved by intrinsic phasing using SHELXT[6] and refined by a full matrix least squares technique based on F^2 using SHELXL2014.[7] The compound crystallised as colourless prisms from acetonitrile. The compound crystallised in a triclinic cell and was solved in the $P\bar{1}$ space group, with two imidazolium cations and two chloride anions in the asymmetric unit. All non-hydrogen atoms were located in the Fourier Map and refined anisotropically. All carbon bound hydrogen atoms were placed in calculated positions and refined isotropically using a "riding model". All nitrogen bound hydrogen atoms were located in the Fourier Map and refined isotropically. Data and structure refinement given in Table 1 and was deposited via the joint CCDC/FIZ Karlsruhe deposition service, deposition number CCDC 1916237.

Table 1 Crystal data and structure refinement for intermediate *I*.

Empirical formula	$C_7H_{14}ClN_3$
Formula weight	175.66
Temperature/K	119.99(13)
Crystal system	triclinic
Space group	P-1
a/Å	8.7847(3)
b/Å	10.5084(3)
c/Å	10.8699(2)
α/°	86.562(2)
β/°	79.455(2)
γ/°	80.992(3)
Volume/Å^3	973.82(5)
Z	4
ρ_{calc}g/cm^3	1.198
μ/mm^{-1}	3.039
F(000)	376.0
Crystal size/mm^3	0.37 × 0.22 × 0.09
Radiation	CuKα (λ = 1.54184)
2Θ range for data collection/°	8.278 to 147.412
Index ranges	$-10 \leq h \leq 10$, $-13 \leq k \leq 12$, $-12 \leq l \leq 8$
Reflections collected	10652
Independent reflections	3677 [R_{int} = 0.0245, R_{sigma} = 0.0245]
Data/restraints/parameters	3677/0/237
Goodness-of-fit on F^2	1.036
Final R indexes [I>=2σ (I)]	R_1 = 0.0290, wR_2 = 0.0752
Final R indexes [all data]	R_1 = 0.0313, wR_2 = 0.0774
Largest diff. peak/hole / e Å^{-3}	0.29/-0.28

4. General procedure for preparation of 5-*tert*-Butyl-2-[{phenylcarbamoyl}amino]-1*H*-imidazol-3-ium acid salts

Hydrochloride salt (1-H$^+$) (5-*tert*-Butyl-2-[{phenylcarbamoyl}amino]-1*H*-imidazol-3-ium chloride)

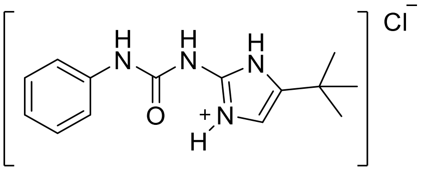

3-(5-tert-Butyl-1H-imidazol-2-yl)-1-phenylurea was dissolved in 4M hydrochloride in 1,4-dixone (1-4 equivalents) and stirred for 1 hour. The solution was concentrated under pressure and dried to give 5-*tert*-Butyl-2-[{phenylcarbamoyl}amino]-1*H*-imidazol-3-ium chloride.^{1}H-NMR (500 MHz, $CDCl_3$): δ 12.56 (br. s, 1H, N*H*), 12.34 (br. s, 1H, N*H*), 11.45 (br. s, 1H, N*H*), 10.09 (s, 1H, N*H*), 7.52 (d, *J* 7.5, 2H, Ar-*H*), 7.29 (t, *J* 7.9, 2H, Ar-*H*), 7.29 (t, *J* 7.5, 1H, Ar-*H*), 6.39 (s, 1H, imidazole-*H*), 1.30 (s, 9H, CH_3 (C^{t}Bu)) ppm.

Trifluoroacetate salt (1-H$^+$) (5-*tert*-Butyl-2-[{phenylcarbamoyl}amino]-1*H*-imidazol-3-ium trifluoroacetate)

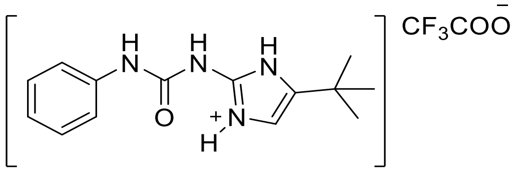

3-(5-tert-Butyl-1H-imidazol-2-yl)-1-phenylurea was dissolved in chloroform (2-10 mM). Trifluoroacetic acid (1 equivalent) was added to give 5-*tert*-Butyl-2-[{phenylcarbamoyl}amino]-1*H*-imidazol-3-ium trifluoroacetate . ^{1}H-NMR (500 MHz, $CDCl_3$): δ 13.28 (br. s, 1H, N*H*), 12.12 (br. s, 1H, N*H*), 8.09 (s, 1H, N*H*), 7.45 (d, *J* 7.9, 2H, Ar-*H*), 7.34 (t, *J* 7.5, 2H, Ar-*H*), 7.14 (t, *J* 7.5, 1H, Ar-*H*), 6.46 (s, 1H, imidazole-*H*), 1.31 (s, 9H, CH_3 (C^{t}Bu)) ppm.

Hexafluorophosphate salt (1-H$^+$) (5-*tert*-Butyl-2-[{phenylcarbamoyl}amino]-1*H*-imidazol-3-ium hexafluorophosphate)

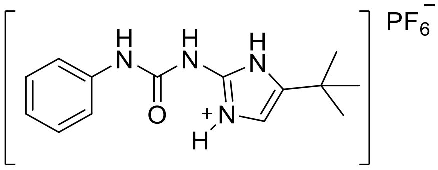

5-*tert*-Butyl-2-[{phenylcarbamoyl}amino]-1*H*-imidazol-3-ium chloride was dissolved in chloroform (2-10 mM). Silver hexafluorophosphate (1 equivalent) was added and stirred for one hour at room temperature. The resultant precipitate was filtered, and the filtrate was concentrated under reduced pressure to give 5-*tert*-Butyl-2-[{phenylcarbamoyl}amino]-1*H*-imidazol-3-ium hexafluorophosphate. ^{1}H-NMR (500 MHz, $CDCl_3$): δ 12.48 (br. s, 1H, N*H*), 12.19 (br. s, 1H, N*H*), 11.57 (br. s, 1H, N*H*), 7.80 (s, 1H, N*H*), 7.43 (d, *J* 7.5, 2H, Ar-*H*), 7.32 (t, *J* 7.5, 2H, Ar-*H*), 7.12 (t, *J* 7.5, 1H, Ar-*H*), 6.42 (s, 1H, imidazole-*H*), 1.31 (s, 9H, CH_3 (C^{t}Bu)) ppm.

5. General procedure for sample preparation for NMR switching experiments

Condition A

The mass of each component was calculated to make a final concentration of 5 mM in 0.6 mL of $CDCl_3$. The required mass of the starting component(s) was dissolved in 0.6 mL of $CDCl_3$. The sample was allowed to equilibrate for a minimum of ten minutes before acquisition. After acquisition the sample was protonated by the addition of 1 equivalent of 4M HCl in 1,4-dioxane solution directly to the sample tube. The sample was allowed to equilibrate for a minimum of ten minutes before acquisition. After acquisition the sample was transferred to a vial and deprotonated by the addition of excess basic $NaHCO_3$ solution. The aqueous layer was separated, and the organic layer was dried and added to an NMR sample tube. The sample was allowed to equilibrate for a minimum of ten minutes before acquisition. Any additional components were added to the same sample when required and the protonation and deprotonation method was repeated as required.

Condition B

The mass of each component was calculated to make a final concentration of 5 mM in 0.6 mL of $CDCl_3$. The required mass of the starting component(s) was dissolved in 0.6 mL of $CDCl_3$. The sample was allowed to equilibrate for a minimum of ten minutes before acquisition. After acquisition the sample was protonated by the addition of 1 equivalent of TFA directly to the sample tube. The sample was allowed to equilibrate for a minimum of ten minutes before acquisition. After acquisition the sample was deprotonated by the addition of 1 or 3 equivalents of DABCO directly to the sample tube. The sample was allowed to equilibrate for a minimum of ten minutes before acquisition. Any additional components were added to the same sample when required and the protonation and deprotonation method was repeated as required.

6. Spectral Data

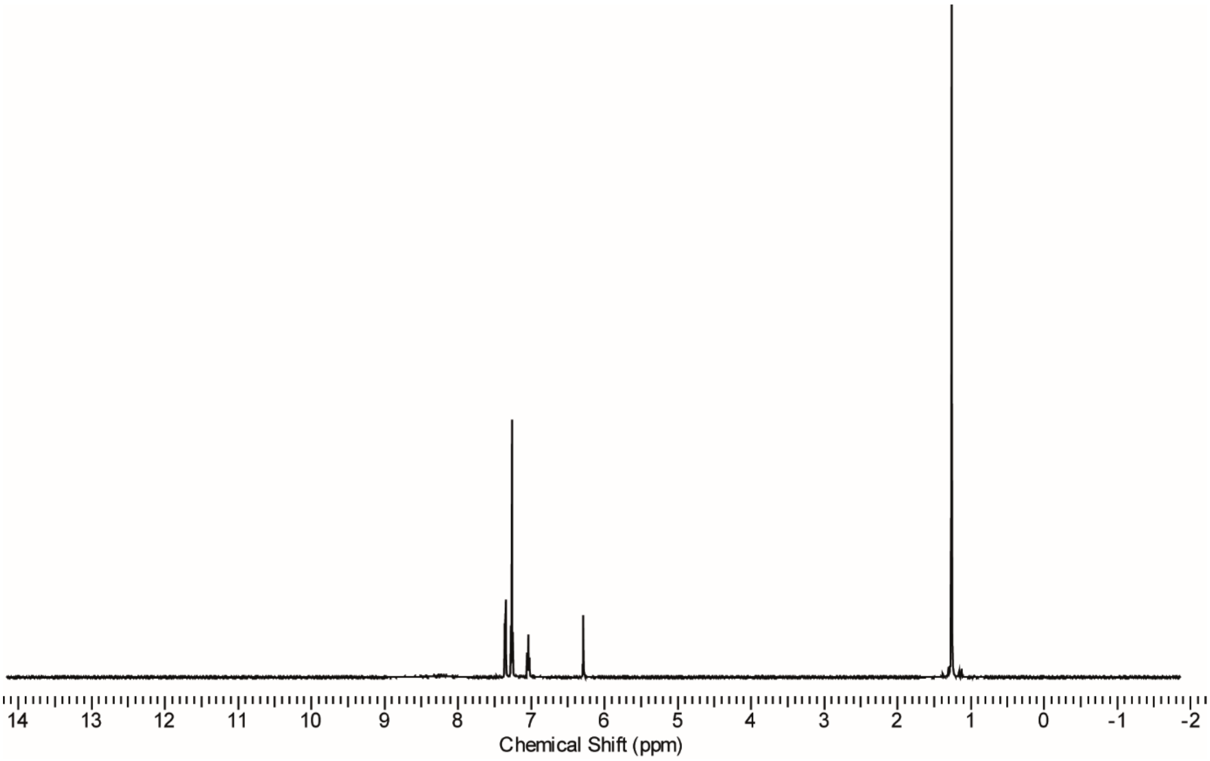

^{1}H NMR UIM **1** (*500 MHz,* 10 mM, $CDCl_3$)

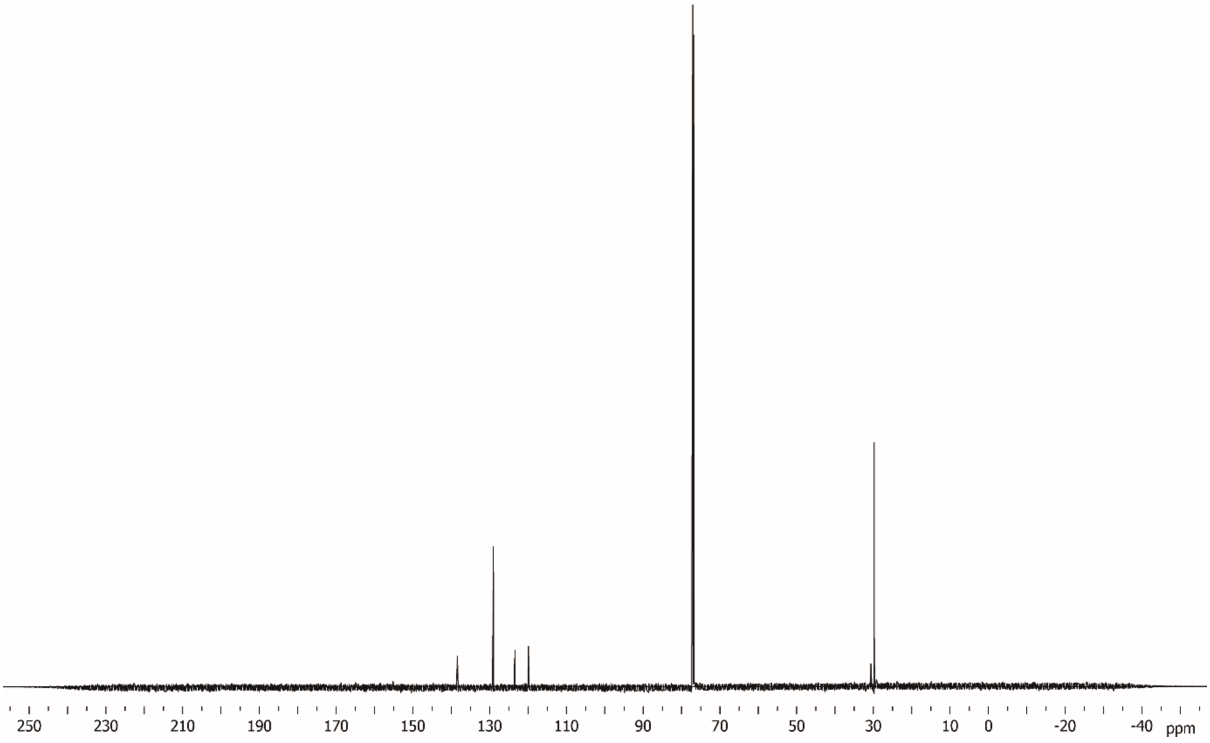

^{13}C NMR UIM **1** (*150 MHz,* 30 mM, $CDCl_3$)

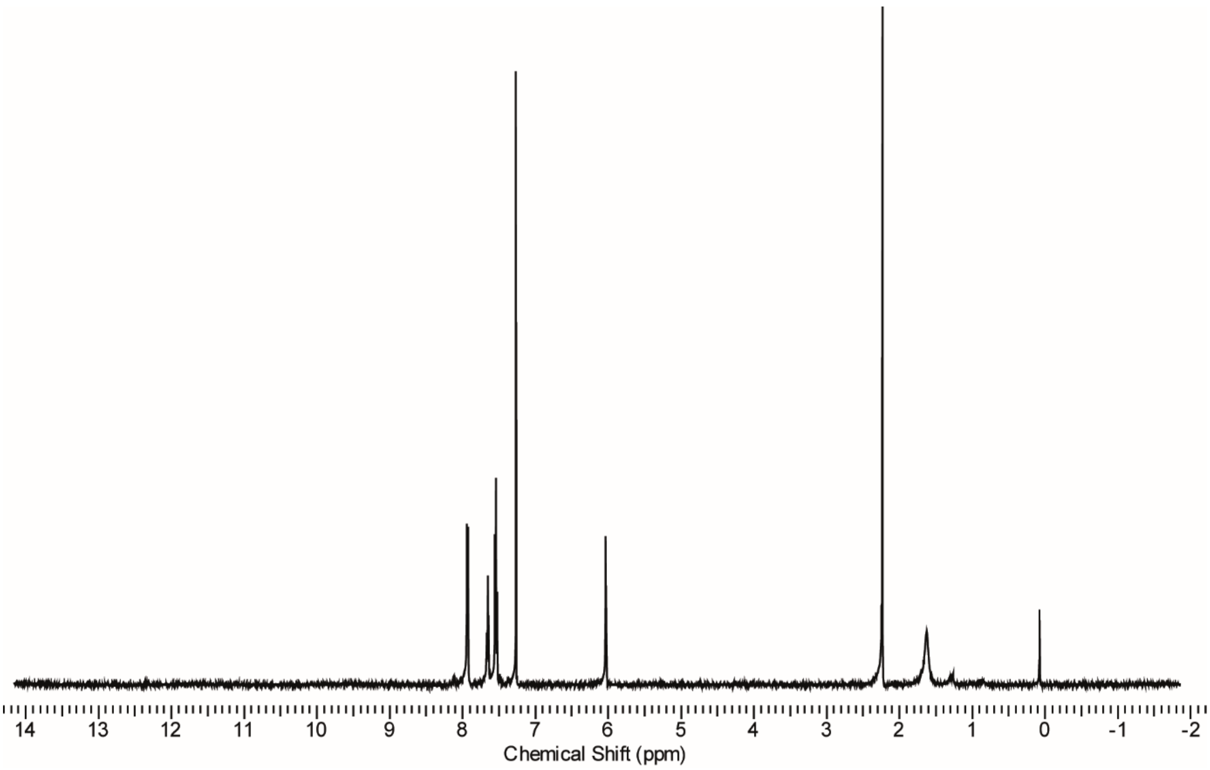

^{1}H NMR AIC **2** (*500 MHz*, 10 mM, $CDCl_3$)

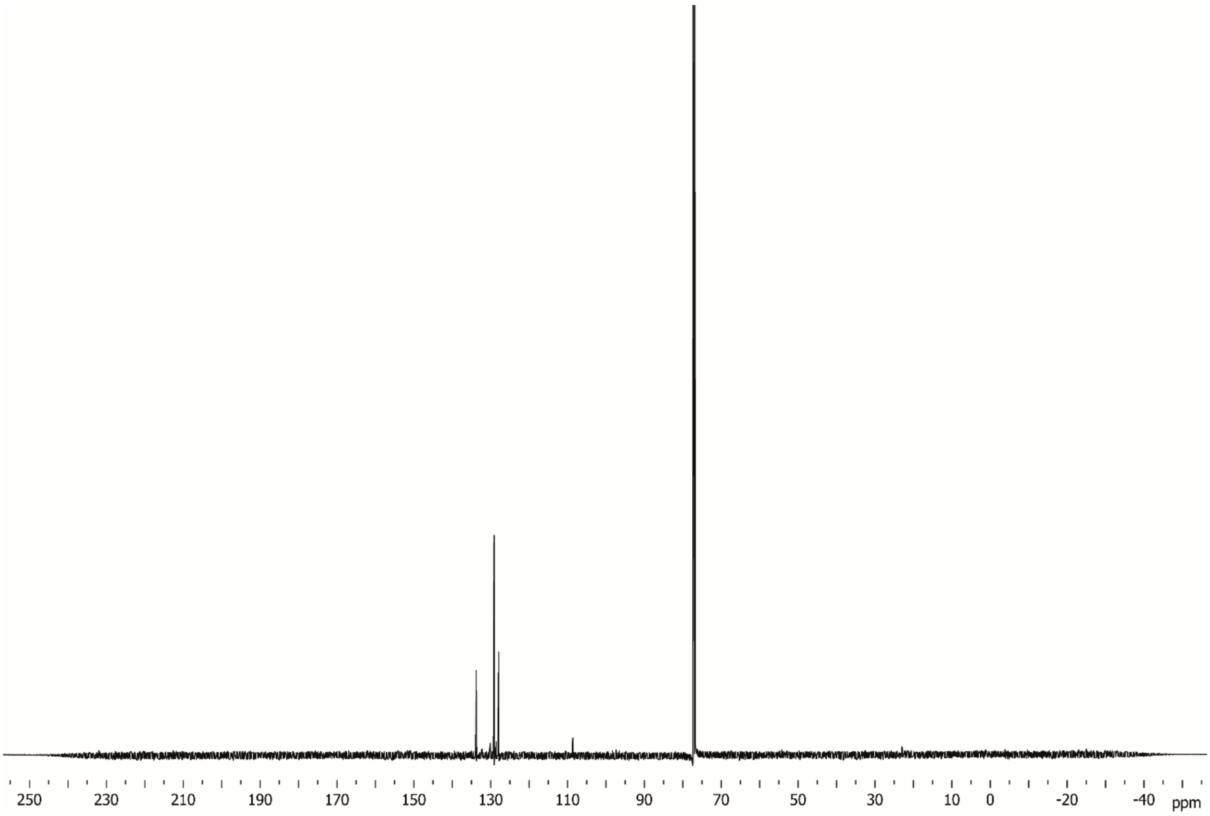

^{13}C NMR AIC **2** (*150 MHz*, 30 mM, $CDCl_3$)

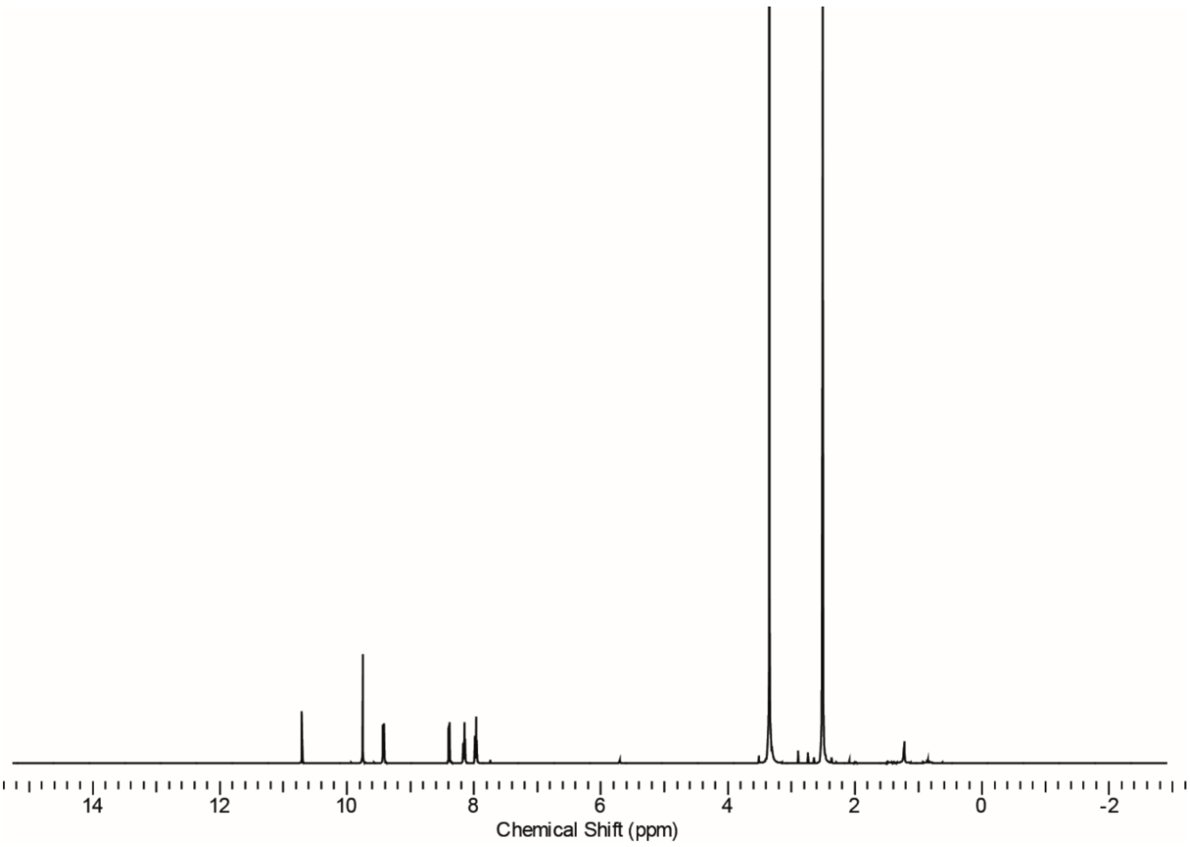

^{1}H NMR BB1 **3** (*500 MHz*, 10 mM, DMSO)

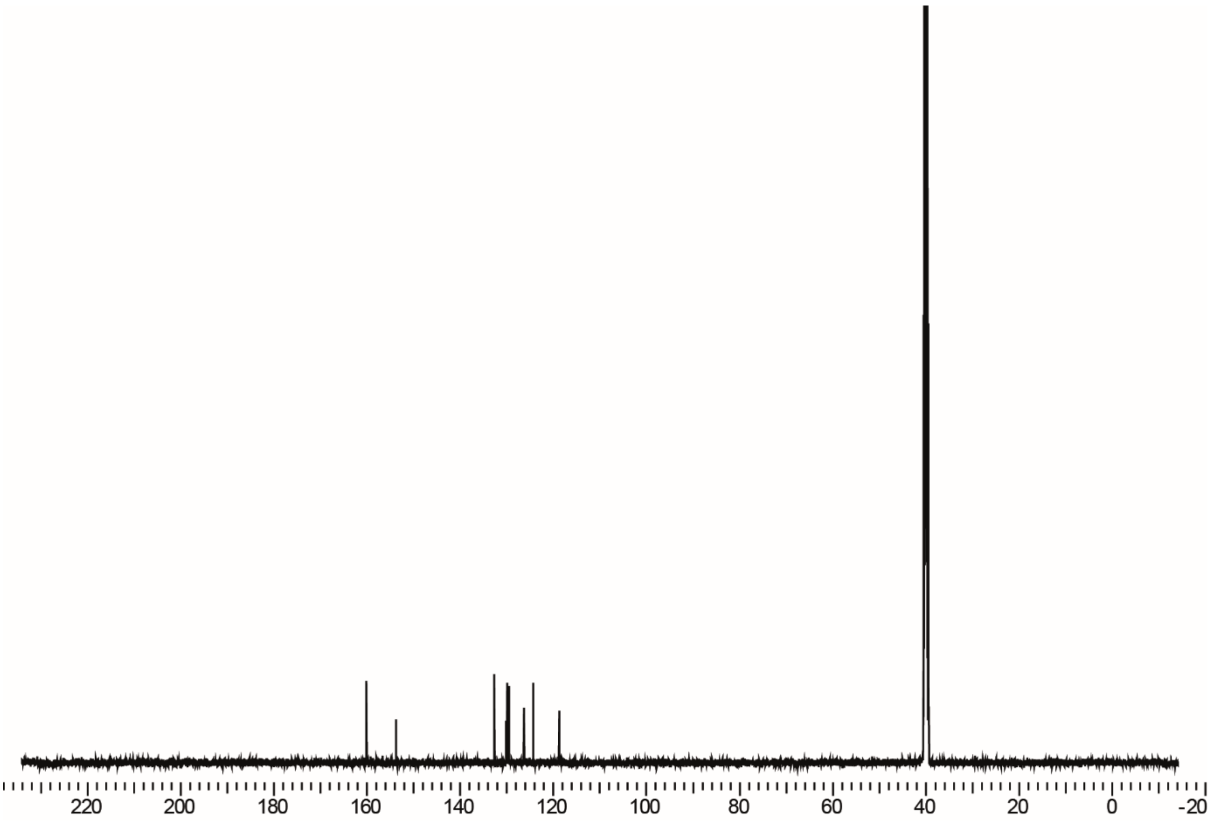

^{13}C NMR BB1 **3** (*125 MHz*, 10 mM, DMSO)

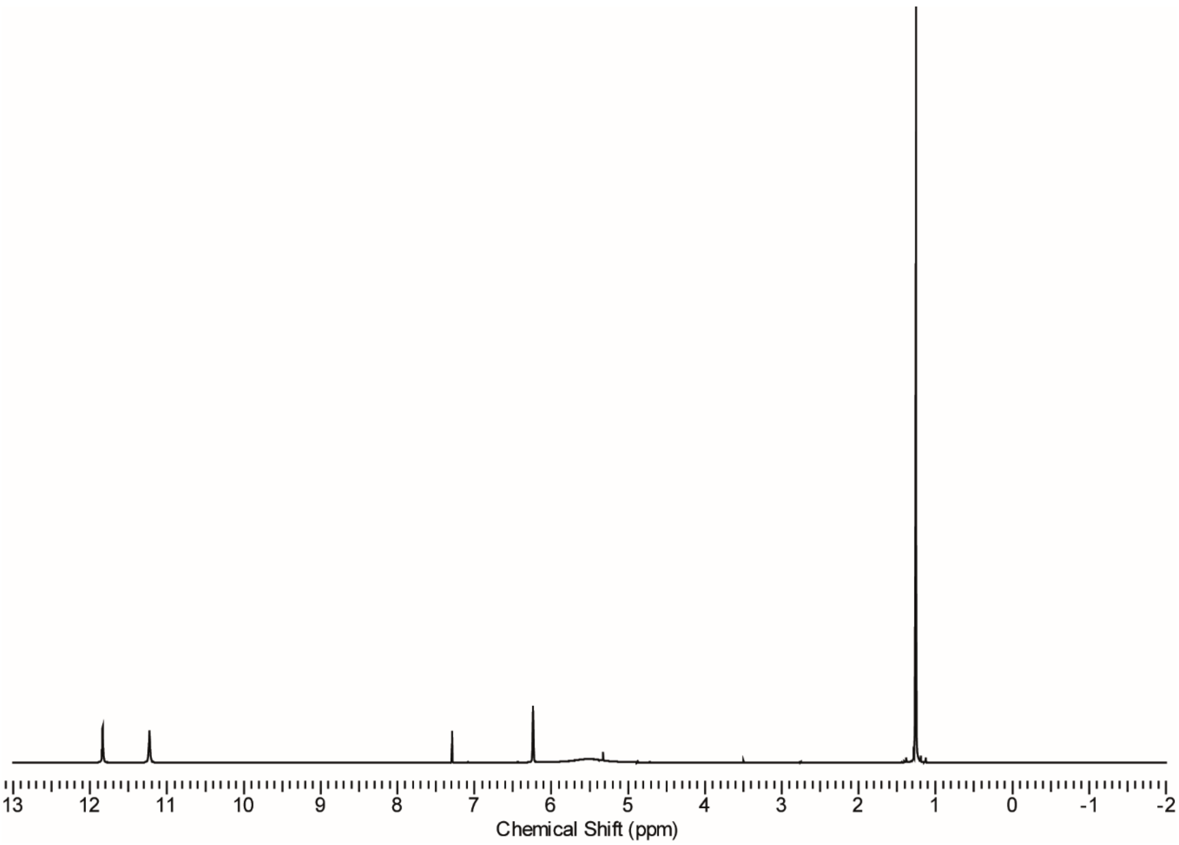

^{1}H NMR ***I*** (*500 MHz*, 10 mM, $CDCl_3$)

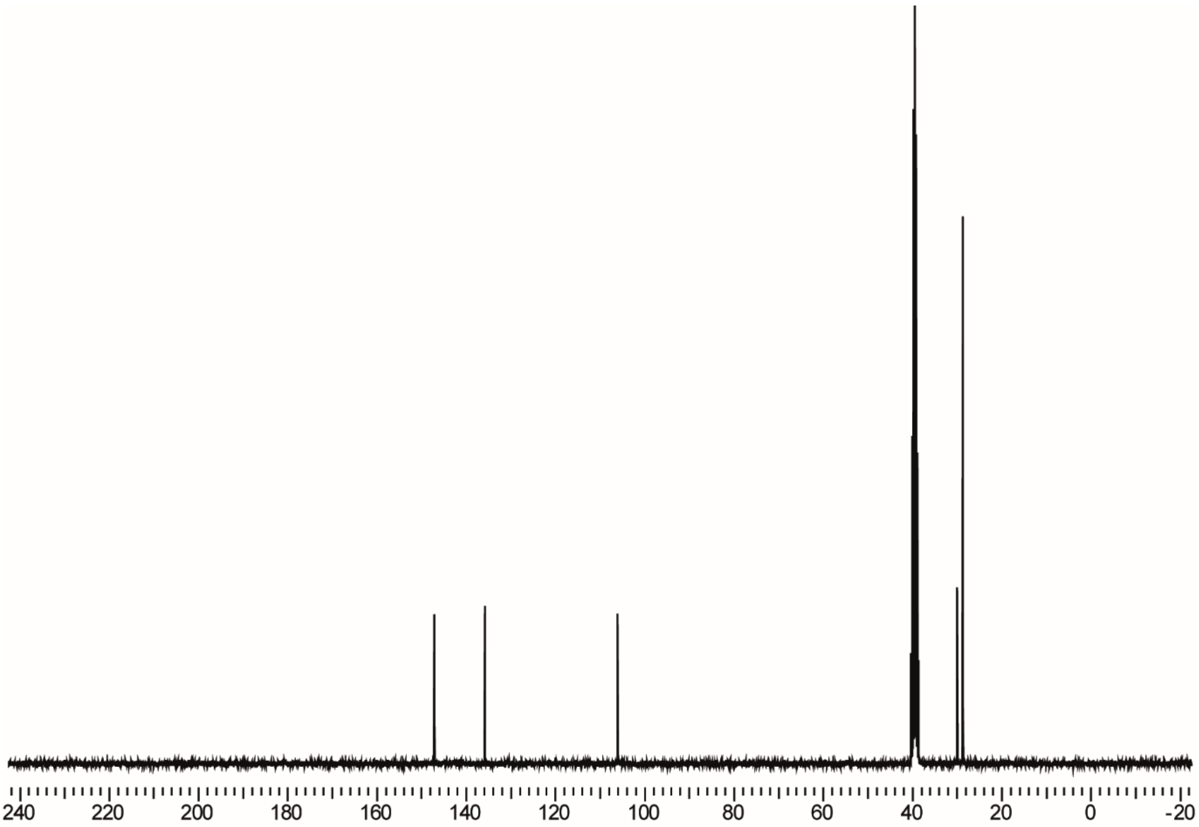

^{13}C NMR ***I*** (*125 MHz*, 10 mM, DMSO)

7. References

[1] “http://supramolecular.org,” **n.d.**

[2] D. Brynn Hibbert, P. Thordarson, *Chem. Commun.* **2016**, *52*, 12792–12805.

[3] H. M. Coubrough, S. C. C. van der Lubbe, K. Hetherington, A. Minard, C. Pask, M. J. Howard, C. Fonseca Guerra, A. J. Wilson, *Chem. – A Eur. J.* **2019**, *25*, 785–795.

[4] C. Koradin, W. Dohle, A. L. Rodriguez, B. Schmid, P. Knochel, *Tetrahedron* **2003**, *59*, 1571–1587.

[5] B. A. Blight, A. Camara-Campos, S. Djurdjevic, M. Kaller, D. A. Leigh, F. M. McMillan, H. McNab, A. M. Z. Slawin, *J. Am. Chem. Soc.* **2009**, *131*, 14116–14122.

[6] G. M. Sheldrick, *Acta Crystallogr. Sect. A* **2015**, *71*, 3–8.

[7] G. M. Sheldrick, *Acta Crystallogr. Sect. C* **2015**, *71*, 3–8.
